# Evaluation of fluoropyruvate as nucleophile in reactions catalysed by *N*-acetyl neuraminic acid lyase variants: scope, limitations and stereoselectivity†
†Electronic supplementary information (ESI) available. See DOI: 10.1039/c5ob02037a



**DOI:** 10.1039/c5ob02037a

**Published:** 2015-11-05

**Authors:** Jennifer Stockwell, Adam D. Daniels, Claire L. Windle, Thomas A. Harman, Thomas Woodhall, Tomas Lebl, Chi H. Trinh, Keith Mulholland, Arwen R. Pearson, Alan Berry, Adam Nelson

**Affiliations:** a School of Chemistry , University of Leeds , Leeds , LS2 9JT , UK . Email: a.s.nelson@leeds.ac.uk; b Astbury Centre for Structural Molecular Biology , University of Leeds , Leeds , LS2 9JT , UK . Email: a.berry@leeds.ac.uk; c School of Molecular and Cellular Biology , University of Leeds , Leeds , LS2 9JT , UK; d School of Chemistry , University of St Andrews , St Andrews , KY16 9ST , UK; e Chemical Development , AstraZeneca , Silk Road Business Park , Macclesfield , Cheshire , SK10 2NA , UK

## Abstract

The catalysis of reactions involving fluoropyruvate as donor by *N*-acetyl neuraminic acid lyase (NAL) variants was investigated. Under kinetic control, the wild-type enzyme catalysed the reaction between fluoropyruvate and *N*-acetyl mannosamine to give a 90 : 10 ratio of the (3*R*,4*R*)- and (3*S*,4*R*)-configured products; after extended reaction times, equilibration occurred to give a 30 : 70 mixture of these products. The efficiency and stereoselectivity of reactions of a range of substrates catalysed by the E192N, E192N/T167V/S208V and E192N/T167G NAL variants were also studied. Using fluoropyruvate and (2*R*,3*S*)- or (2*S*,3*R*)-2,3-dihydroxy-4-oxo-*N*,*N*-dipropylbutanamide as substrates, it was possible to obtain three of the four possible diastereomeric products; for each product, the ratio of anomeric and pyranose/furanose forms was determined. The crystal structure of *S. aureus* NAL in complex with fluoropyruvate was determined, assisting rationalisation of the stereochemical outcome of C–C bond formation.

## Abstract 2

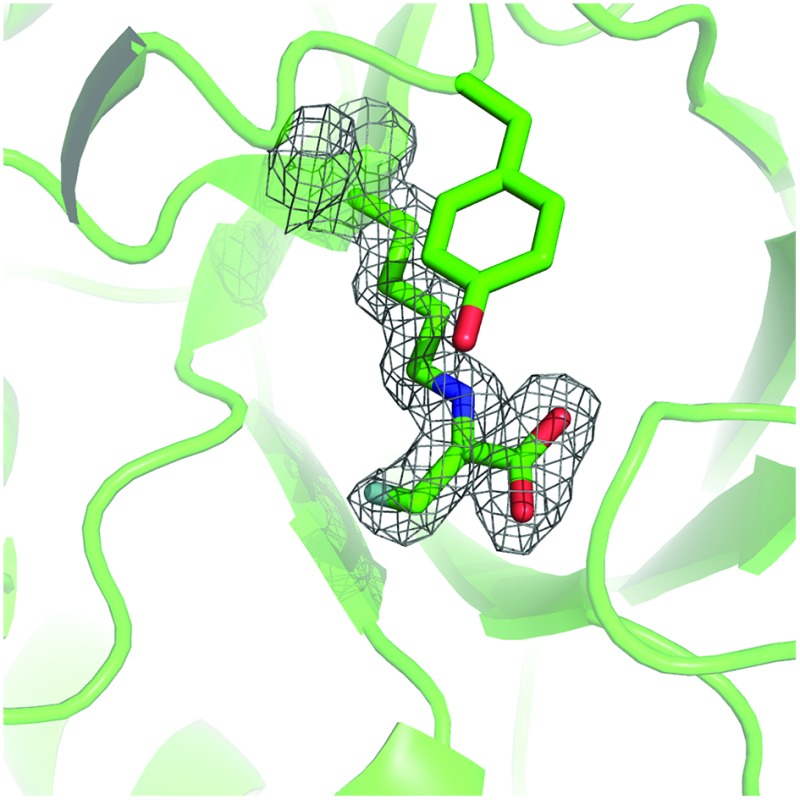
The stereochemical course of aldolase-catalysed reaction between fluoropyruvate and aldehydes is described.

## Introduction

**Fig. 1:**
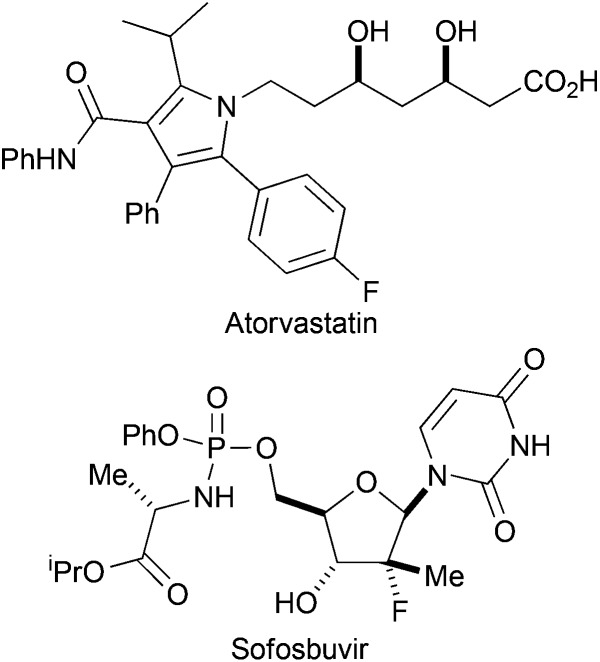
Examples of fluorinated drugs.

The introduction of fluorine can have a profound effect on bioactive molecules including their conformation, binding, bioavailability, metabolism, pharmacokinetics and pharmacodynamics.^1^ As a consequence, around 20% of prescribed drugs, and 30% of leading blockbuster drugs, contain at least one fluorine atom.^2^ Examples of fluorinated pharmaceuticals include the cholesterol-lowering drug Atorvastatin, and Sofosbuvir which is exploited in the treatment of Hepatitis C (Fig. 1).^3,4^ Moreover, fluorinated sugars can serve as valuable mechanism-based probes of carbohydrate-processing enzymes.^5^

The stereoselective synthesis of compounds with a fluorine-bearing stereocentre is a significant challenge. Most solutions to this problem rely on stereoselective C–F bond formation, for example by fluorination of allylic silanes.^6^ Some catalytic methods for enantioselective C–F bond formation have been developed: for example by organocatalytic α-fluorination of aldehydes^7^ or Pd-catalysed α-fluorination of β-keto phosphonates.^8^

**Scheme 1:**
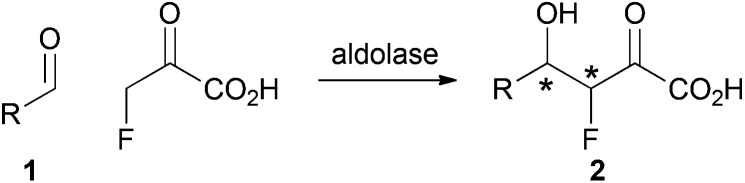
Envisaged strategy for controlling F-bearing stereocentres by C–C bond formation.

We envisaged a complementary catalytic approach in which a F-bearing stereocentre would be controlled by formation of a neighbouring C–C bond (Scheme 1). Aldolase-catalysed reaction involving fluoropyruvate and an aldehyde **1** would yield an aldol product **2** with two new stereogenic centres. This catalytic approach would complement enantioselective aldol reactions involving fluoroacetone.^9^

*N*-Acetyl neuraminic acid lyase (NAL) is a Class I aldolase that catalyses the reversible aldol reaction between pyruvate and *N*-acetyl mannosamine (ManNAc) to give *N*-acetyl neuraminic acid (Neu5Ac). A combination of mutagenesis, structural biology and computational chemistry has revealed insights into its catalytic mechanism.^10^ Despite a report that it is not a substrate,^11^ fluoropyruvate is a viable donor.^12^ However, differing stereochemical outcomes have been reported for the NAL-catalysed reaction between fluoropyruvate and ManNAc.^12^ An initial aim of our study was, therefore, to clarify the stereochemical outcome of this reaction.

**Scheme 2:**
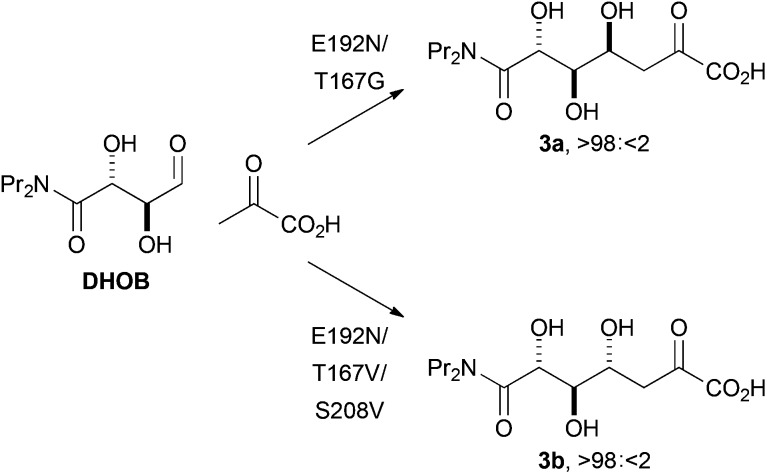
Stereoselective aldol reactions catalysed by aldolases generated by directed evolution.^14^ The products are drawn in open chain form for clarity.

We also sought to investigate the catalysed reactions between fluoropyruvate and alternative aldehyde acceptors. Here, we investigated the value of synthetically-useful NAL variants that we have previously generated using directed evolution.^13,14^ The E192N variant of NAL is an excellent catalyst of the poorly stereoselective reaction between pyruvate and the alternative substrate (2*R*,3*S*)-2,3-dihydroxy-4-oxo-*N*,*N*-dipropylbutanamide, **DHOB** (Scheme 2).^13^ The structural basis of the modified substrate specificity of this variant has been gleaned using protein crystallography.^15^ In contrast, the E192N/T167G and E192N/T167V/S208V variants of NAL control the stereochemistry of C–C bond formation, and catalyse respectively the selective formation of the alternative diastereomeric products **3a** and **3b** (Scheme 2).^14^

## Results and discussion

### Evaluation of wild-type NAL in the synthesis of fluorinated analogues of *N*-acetyl neuraminic acid

**Scheme 3:**
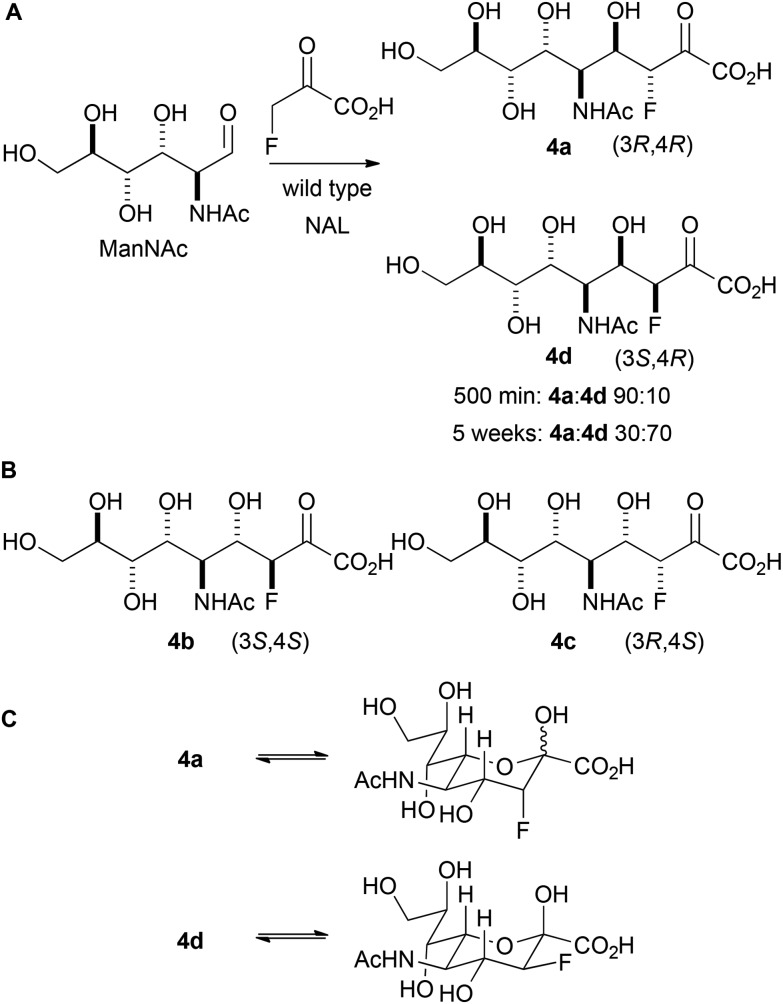
Reaction between fluoropyruvate and ManNAc catalysed by wild-type NAL. A: The stereochemical outcome is determined by the reaction time. The products are depicted in open chain form for clarity. NAL was regularly added to the 5 week reaction. B: Diastereomeric products that were not observed. C: Cyclised forms of **4a** and **4d**.

Initially, the reaction between fluoropyruvate and ManNAc catalysed by wild-type NAL was investigated (Panel A, Scheme 3). Accordingly, the reaction was performed at 37 °C in an NMR tube (20 mM sodium fluoropyruvate and 100 mM *N*-acetyl mannosamine in 20 mM Tris-HCl pH 7.4 buffer) and followed by ^19^F NMR spectroscopy. After 500 min, the fluoropyruvate was >98% consumed, and a 90 : 10 mixture of the diastereomeric products **4a** and **4d** had been formed (which vary only in their configuration at C-3). However, after a prolonged reaction time (∼5 weeks), with regular addition of more enzyme, the ratio of products had switched to 30 : 70 in favour of **4d**. The alternative possible diastereomeric products (**4b** and **4c**; Panel B) were not detected. These data suggest that **4a** is the kinetic product of the reaction, and that **4d** is the thermodynamic product. The relative thermodynamic stability of **4d** may stem from the stabilising gauche interaction between fluorine and vicinal electronegative atoms.^16^ As with previous studies, the reaction was found to yield selectively (4*R*)-configured products. The different ratios of products under kinetic and thermodynamic control may account for the contrasting selectivities reported in previous studies.^12^

The interpretation of the spectroscopic data was greatly assisted by the preparation of standard samples of the products **4a** and **4d**.^12*a*^ The reaction between ManNAc and sodium fluoropyruvate, catalysed by wild-type NAL, was conducted at 37 °C in 100 mM Tris-HCl pH 7.4 buffer, and the products purified by column chromatography. After 24 h reaction, the product **4a** was obtained in 34% yield; whilst after reaction for >1 week, the product **4d** was obtained in 43% yield. In both cases, a single pyranose anomer predominated (Table 1; Panel C, Scheme 3). The configuration of **4a** and **4d** was determined by careful analysis of vicinal coupling constants.^17^ In both pyranose anomers of **4a**, there was a large coupling constant between fluorine and H-4 (∼30 Hz) and a small coupling constant between H-3 and H-4 (2.1 Hz in the major anomer) (Table 1). In contrast, in the major anomer of **4d**, there was a small coupling constant between fluorine and H-4 (∼12 Hz) and a large coupling constant between H-3 and H-4 (8.8 Hz).

**Table 1:** Spectroscopic data for the fluorinated products of aldolase-catalysed reactions

Product	Form (proportion)	*δ*_F_/ppm	*δ*_3H_/ppm	*δ*_4H_/ppm	*δ*_5H_/ppm	*δ*_6H_/ppm	^2^ *J*_HF_ ^*a*^/Hz	^3^ *J*_HF_ ^*a*^/Hz	^3^ *J*_3H–4H_/Hz	^3^ *J*_4H–5H_/Hz	^3^ *J*_5H–6H_/Hz
**4a**	Major pyranose (98%)	–208.1	4.65	3.93	4.07	3.87	49.3	30.0	2.1	10.6	10.6
	Minor pyranose (2%)	–217.9	NM^*b*^	NM^*b*^	NM^*b*^	NM^*b*^	51.3	29.9	NM^*b*^	NM^*b*^	NM^*b*^
**4d**	Major pyranose (96%)	–199.3	4.47	∼3.90	∼3.90	∼3.90	49.7	12.0	8.8	NM^*b*^	NM^*b*^
**16a**	Major pyranose (92%)	–206.0	4.78	3.94	3.88	4.75	49.9	32.5	3.4	9.7	9.2
	Minor pyranose (8%)	–216.8	NM^*b*^	NM^*b*^	NM^*b*^	NM^*b*^	51.4	32.8	NM^*b*^	NM^*b*^	NM^*b*^
**16c**	Major pyranose (35%)	–190.5	4.85	4.39	4.12	4.65	50.5	24.0	4.8	5.0	6.1
	Major furanose (25%)	–194.5	4.72	4.02	3.96	NM^*b*^	43.7	4.7	1.7	NM^*b*^	NM^*b*^
	Minor pyranose (30%)	–201.9	5.03	4.46	3.95	4.57	53.1	18.7	5.5	5.6	7.2
	Minor furanose (10%)	–207.4	4.86	4.30	4.18	4.75	48.5	10.1	7.3	NM^*b*^	5.4
*ent*-**16d**	Pyranose (>98%)	–199.8	4.60	3.95	3.78	4.62	49.3	13.3	9.3	9.3	9.7
**17a**	Major pyranose (98%)	–207.8	4.90	4.16	4.23	4.83	49.0	29.1	2.2	10.9	10.0
	Minor pyranose (2%)	–218.5	NM^*b*^	NM^*b*^	NM^*b*^	NM^*b*^	50.2	28.8	NM^*b*^	NM^*b*^	NM^*b*^

^*a*^Determined by analysis of the 296 MHz ^19^F NMR spectrum.

^*b*^Not measured.

The catalysis of the cleavage of the reaction products **4a** and **4d** was also studied using an established coupled enzyme assay^13*b*^ (Table 2). The cleavage of the fluorinated *N*-acetyl neuraminic acid analogue **4a** was much less efficient than that of Neu5Ac itself (*k*_cat_/*K*_M_: 0.11 min^–1^ mM^–1^ for **4a** compared with 260 min^–1^ mM^–1^ for Neu5Ac). However, the catalysis of the cleavage of the diastereomeric fluorinated analogue **4d** was even less efficient and was not detectable under the conditions of the assay. This observation is consistent with (3*R*,4*R*)-configured **4a** being the kinetic product of the NAL-catalysed reaction between fluoropyruvate and ManNAc.

**Table 2:** Kinetic parameters for the cleavage of substrates catalysed by wild-type NAL^*a*^

Substrate	*k*_cat_/min^–1^	*K*_M_/mM	*k*_cat_/*K*_M_/min^–1^ mM^–1^
Neu5Ac	510 ± 10	2.0 ± 0.1	260
**4a**	0.91 ± 0.03	8.4 ± 0.7	0.11
**4b**	ND^*b*^	ND^*b*^	

^*a*^Determined using a coupled enzyme assay involving lactate dehydrogenase.

^*b*^Not detectable.

### Preparation of substrate precursors

**Scheme 4:**
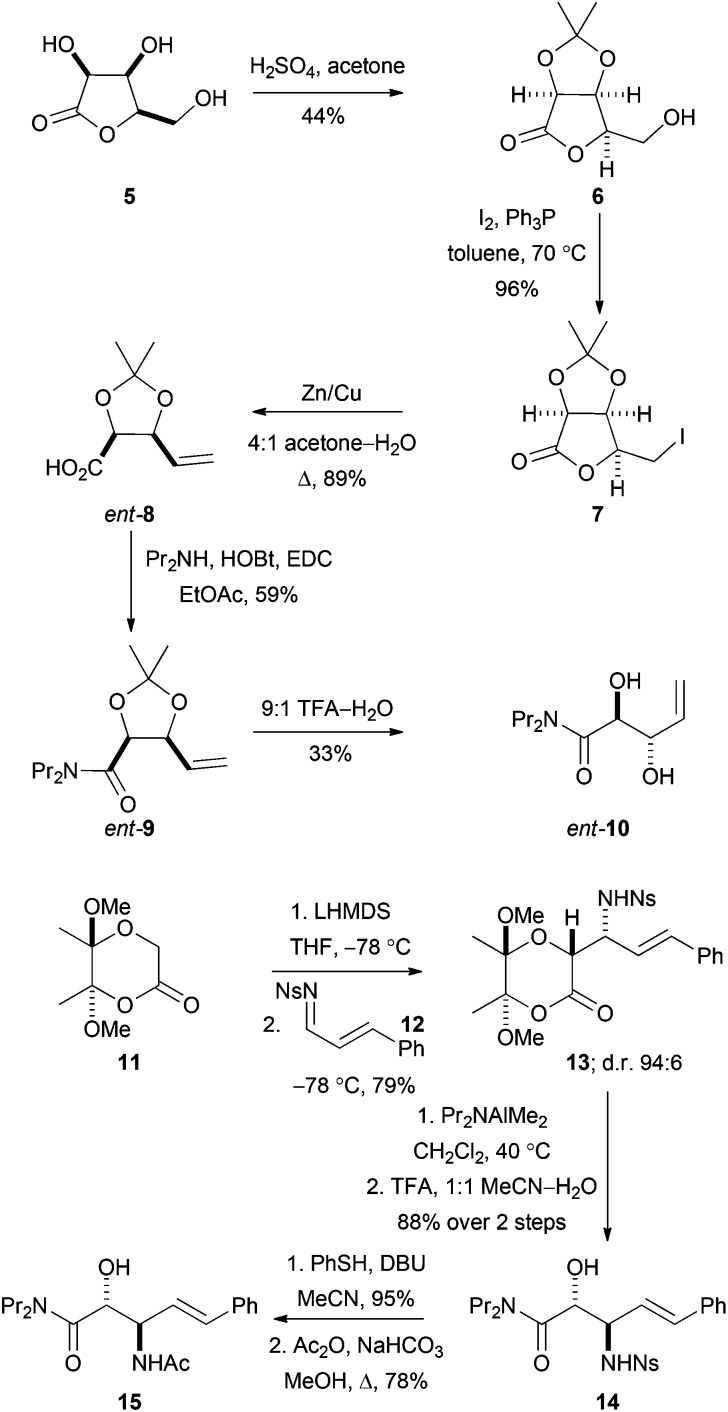
Synthesis of precursors of aldehyde substrates. In addition, the alkene *ent*-**15** was prepared from the enantiomeric lactone starting material **11**; and **10** was prepared using an established route.^16^

To enable evaluation of alternative potential substrates, a range of alkene precursors was prepared: ozonolysis of these alkenes (**10**, *ent*-**10**, **15** and *ent*-**15**) would yield the corresponding aldehydes (**DHOB**, *ent*-**DHOB**, **AHOB** §
§(2*R*,3*S*)-3-acetyl-2-hydroxy-4-oxo-*N*,*N*-dipropylbutanamide. and *ent*-**AHOB**). The alkene^13*a*^ *ent*-**10** was prepared using a route that was analogous to an established^18^ synthesis of **10** (Scheme 4). Thus, treatment of the lactone **5** (derived from lyxose^19^) with concentrated hydrochloric acid in acetone gave the corresponding acetonide^20^ **6**. Treatment of **6** with iodine and triphenylphosphine gave the corresponding iodolactone **7**, which was followed by reductive ring-opening to give the carboxylic acid *ent*-**8** (whose enantiomer had been used to prepare^18^ **10**). Finally, amide formation (→**9**) and deprotection gave the required alkene *ent*-**10**.

The alkenes **15** and *ent*-**15** were prepared from the known^21^ enantiomerically pure lactones **11** and *ent*-**11** (see Scheme 4 for the synthesis of **15**). Treatment of the lactone **11** with LHMDS at –78 °C, and reaction with the *N*-sulfonyl imine **12**, gave the product **13** as a 94 : 6 mixture of diastereomers; the relative configuration of the major diastereomer was determined by subsequent conversion into a cyclic derivative (see below). The lactone **13** was ring-opened by treatment with Pr_2_NAlMe_2_ and, following acetal hydrolysis, the β-amino amide derivative **14** was obtained in 88% yield. Finally, desulfonylation of **14**, followed by acetylation, gave the required alkene **15**.

### Evaluation of variant NALs in the catalysis of reactions involving fluoropyruvate

#### Efficiency of catalysis

**Scheme 5:**
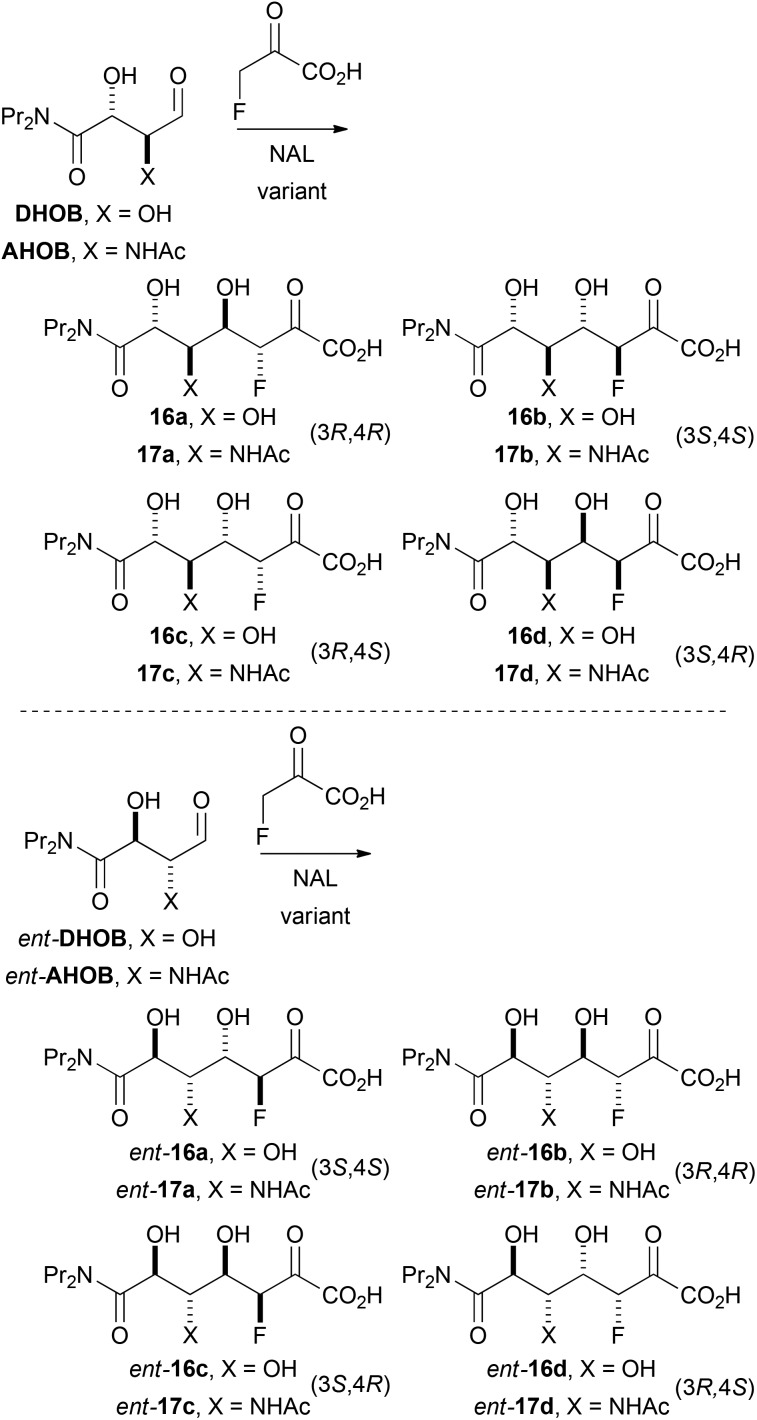
Possible diastereomeric products of aldolase-catalysed reactions with fluoropyruvate as nucleophile.

The ability of NAL variants to catalyse reactions involving fluoropyruvate as donor was investigated. The aldehydes **DHOB**, *ent*-**DHOB**, **AHOB** and *ent*-**AHOH** were investigated as potential substrates for the E192N, E192N/T167G and E192N/T167V/S208 V NAL variants (Scheme 5). The efficiency of catalysis was initially investigated by determining the rate of consumption of fluoropyruvate by ^19^F NMR spectroscopy. In each case, the corresponding alkene precursor (**10**, *ent*-**10**, **15** or *ent*-**16**) was cleaved ozonolytically in methanol at –78 °C, and the reaction mixture quenched with dimethylsulfide, transferred into an NMR tube and evaporated; the reaction (20 mM sodium fluoropyruvate and 100 mM substrate in 50 mM Tris-HClpH 7.4 buffer) to give aldol products was then followed by 296 MHz ^19^F NMR spectroscopy. The results are presented in Table 3.

**Table 3:** Activity and selectivity of aldolase variants with fluoropyruvate as donor

Substrate^*a*^	Variant	Specific activity^*b*^/nmol min^–1^ nmol^–1^	Product	Ratio^*c*^ *a* : *b* : *c* : *d*	(3*R*,4*R*) : (3*S*,4*S*) : (3*R*,4*S*) : (3*S*,4*R*)^*c*^
**DHOB**	E192N	9.1	**16**	40 : 0 : 50 : 10	40 : 0 : 50 : 10
**DHOB**	E192N/T167V/S208V	0.56	**16**	0 : 0 : 100 : 0	0 : 0 : 100 : 0
**DHOB**	E192N/T167G	0.06	**16**	30 : 0 : 70 : 0	30 : 0 : 70 : 0
*ent*-**DHOB**	E192N	0.46	*ent*-**16**	10 : 0 : 0 : 90	0 : 10 : 90 : 0
*ent*-**DHOB**	E192N/T167V/S208V	0.03	*ent*-**16**	0 : 0 : 0 : 100	0 : 0 : 100 : 0
*ent*-**DHOB**	E192N/T167G	0.12	*ent*-**16**	20 : 0 : 0 : 80	0 : 20 : 80 : 0
**AHOB**	E192N	1.1	**17**	60 : 0 : 40 : 0	60 : 0 : 40 : 0
**AHOB**	E192N/T167V/S208V	ND^*e*^	—		
**AHOB**	E192N/T167G	0.03	**17**	NM^*d*^	
*ent*-**AHOB**	E192N	0.07	*ent*-**17**	NM^*d*^	
*ent*-**AHOB**	E192N/T167V/S208V	ND^*e*^	—		
*ent*-**AHOB**	E192N/T167G	0.03	*ent*-**17**	NM^*d*^	

^*a*^Prepared by ozonolysis of the corresponding alkene (**10**, *ent*-**10**, **15** or *ent*-**15**).

^*b*^Consumption of fluoropyruvate (nmol min^–1^ per nmol protein) determined by 296 MHz ^19^F NMR spectroscopy.

^*c*^Kinetic ratio of diastereomeric products determined by 296 MHz ^19^F NMR spectroscopy.

^*d*^Not measured.

^*e*^Not detectable.

The rate of consumption of fluoropyruvate was highest with the combination of **DHOB** and the E192N variant (9.1 nmol min^–1^ per nmol protein). This observation is, perhaps, unsurprising given that E192N was obtained *via* a directed evolution approach that sought to optimise catalysis of cleavage to yield **DHOB**.^13^ However, it is notable that the E192N variant – in addition to the wild-type enzyme – accepts fluoropyruvate as an alternative donor.

Catalysis by the E192N variant was significantly less efficient with the other substrates investigated. For example, with **AHOB**, in which the α-hydroxy group of **DHOB** has been replaced with an α-NHAc group, the rate of consumption of fluropyruvate was about 8-fold slower. Switching to the enantiomeric substrate series was also detrimental to catalysis: the rate of consumption of fluoropyruvate was about 20-fold slower with *ent*-**DHOB** (compared to **DHOB**) and about 15-fold slower with *ent*-**AHOB** (compared to **AHOB**).

In addition, the E192N/T167G and E192N/T167V/S208V variants are less efficient catalysts than the E192N variant. For example, with **DHOB** as substrate, the rate of consumption of fluoropyruvate was about 15- and 150-fold slower with the E192N/T167G and E192N/T167V/S208V variants respectively than with the E192N variant. These variants were generated to catalyse complementary stereoselective reactions between pyruvate and **DHOB** (Scheme 2): a reduction in the efficiency of catalysis (compared to the E192N variant) was also observed with pyruvate as the donor substrate.^14^

### Preparation and characterisation of reaction products

The determination of the stereoselectivity of the reactions was complicated by the possibility of four diastereomeric products, each of which might exist in different anomeric and pyranose/furanose forms. To assist analysis, selected reactions were conducted preparatively, and the products purified and characterised (Table 4). In each case, the aldehyde substrate and sodium fluoropyruvate were dissolved in 50 mM Tris-HCl pH 7.4 buffer, and the relevant NAL variant added. The conversion of each reaction was determined by analysis of the crude product by 296 MHz ^19^F NMR spectroscopy.

**Table 4:** Preparation of fluorinated products of aldolase-catalysed reactions

Substrate^*a*^ (eq.)	Variant	Product^*b*^	Time/day (conversion^*c*^/%)	Yield^*d*^/% (ratio^*e*^)
**DHOB** (2 eq.)	E192N	**16a** and **16c**	2 (>99)	33^*f*^ (40 : 60)
**DHOB** (2 eq.)	E192N/T167 V/S208V	**16c**	2 (95)	41
*ent*-**DHOB** (1 eq.)	E192N/T167 V/S208V	*ent*-**16d**	1 (NM^*g*^)	52
**AHOB** (5 eq.)	E192N	**17a**	5 (50)	7^*h*^

^*a*^Prepared by ozonolysis of the corresponding alkene (**10**, *ent*-**10**, **15** or *ent*-**15**).

^*b*^See Table 1 for details of ratios of anomers and pyranose/furanose forms.

^*c*^Determined by analysis of the crude product by 296 MHz ^19^F NMR spectroscopy.

^*d*^Yield of purified product based on the limiting reactant.

^*e*^Determined by 296 MHz ^19^F NMR spectroscopy.

^*f*^Small samples of each diastereomer could be obtained by reverse-phase HPLC.

^*g*^Not measured.

^*h*^After purification by mass-directed HPLC.

In two cases, the aldolase-catalysed reactions were highly diastereoselective, and >98 : <2 mixtures of diastereomeric products were obtained after ion exchange chromatography. Thus, with the E192N/T167V/S208V variant, fluoropyruvate and **DHOB** reacted to give **16c** which was isolated in 41% yield. Similarly, with the same NAL variant, fluoropyruvate and *ent*-**DHOB** reacted to give *ent*-**16d** which was isolated in 52% yield. However, with the E192N variant, fluoropyruvate and **DHOB** were converted into a 40 : 60 mixture of **16a** and **16c** from which it was possible to obtain small samples of both products after reverse-phase HPLC purification. Similarly, using E192N, fluoropyruvate and **AHOB** reacted to give a diastereomeric mixture of products, from which a small sample of **17a** could be obtained by mass-directed HPLC.

**Fig. 2:**
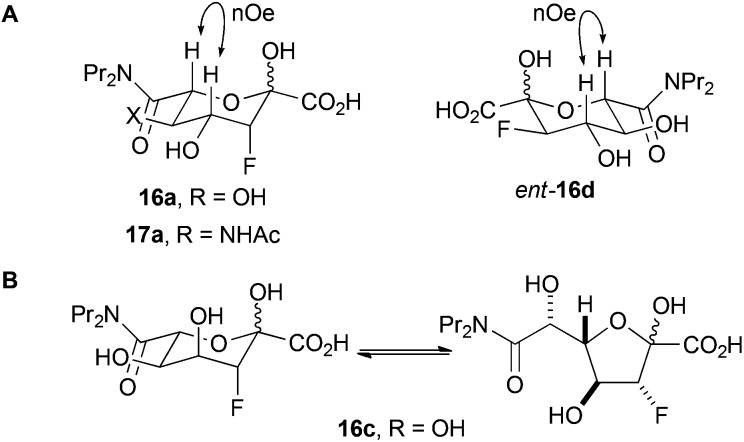
Forms of reaction products. Panel A: The products **16a**, *ent*-**16d** and **17a** exist predominantly in pyranose forms. Panel B: the product **16c** exists as a mixture of pyranose and furanose anomers.

The fluorinated products **16a**, *ent*-**16d** and **17a** existed in pyranose forms (Panel A, Fig. 2). In both pyranose anomers of **16a** and **17a**, there was a large coupling constant between fluorine and H-4 (∼30 Hz); in addition, in the major anomer of each compound, there was a small coupling constant between H-3 and H4 (**16a**: 3.4 Hz; **17a**: 2.2 Hz) (Table 3). These data imply that **16a** and **17a** are (3*R*,4*R*)-configured (and indirectly enabled determination of the relative configuration of **13**). In contrast, *ent*-**16d** had a small coupling constant between the equatorially-positioned fluorine and H-4 (13.3 Hz) and a large coupling constant between the axial protons H-3 and H-4 (9.3 Hz). The configuration of **16a** and *ent*-**16d** was corroborated by the observation of nOe interactions between the axial protons at H-4 and H-6. The analysis of **16c** was hugely complicated by the existence of both pyranose and furanose anomers (Panel B, Fig. 2); however, ^1^H/^19^F HSQC-TOCSY spectroscopy enabled extraction of the ^1^H NMR spectra of each of the four species that were present (Table 1 and ESI†). The pyranose anomers of **16c** have axially-oriented fluorine and 4-OH groups which cannot enjoy a stabilising gauche interaction.^16^

#### Stereoselectivity of reactions

The characterisation of the products enabled determination of the stereoselectivity of reactions catalysed by NAL variants (Table 3). With the E192N variant, **DHOB** and fluoropyruvate yielded a 40 : 0 : 50 : 10 mixture of **16a**, **16b**, **16c** and **16d**; this poor stereoselectivity parallels that observed with this enzyme in the reaction between pyruvate and **DHOB**.^13a,14^ In contrast, the E192N/T167V/S208V variant yielded selectively the (3*R*,4*S*)-configured product **16c**. This variant was generated^14^ by directed evolution to yield selectively the 4*S*-configured product (**3b**) with pyruvate as nucleophile (Scheme 2): it is remarkable that the 4*S* selectivity is retained with an alternative nucleophile (fluoropyruvate). However, in contrast, selectivity for 4*R*-configured products was not observed with the E192N/T167G variant: with this variant, the reaction between **DHOB** and fluoropyruvate was very inefficient, and a 30 : 70 mixture of **16a** and **16c** was obtained.

The effect of the structure of the aldehyde substrate on stereoselectivity was also investigated. **AHOB** has an α-NHAc group in place of the α-hydroxy group of **DHOB**; with **AHOB** and the E192N variant, similarly poor stereoselectivity was also observed with this substrate (**17a** : **17b** : **17c** : **17d** 60 : 0 : 40 : 0). In the enantiomeric series, *ent*-**DHOB** gave predominantly the (3*R*,4*S*)-configured product *ent*-**16d** with all three NAL variants; here, the NAL variant had only a small effect on the stereoselectivity of the aldol reaction.

#### Structural insights into stereoselectivity

It is notable that all combinations of substrates and NAL variants yielded 3*R*-configured products selectively under kinetic control. With **DHOB**/**AHOB**, the products **16a**/**17a** [with (3*R*,4*R*) configuration] and/or **16c**/**17c** [with (3*R*,4*S*) configuration] predominated. With *ent*-**DHOB**, although a different diastereomer (*ent*-**16d**) was formed selectively, its absolute configuration was still (3*R*,4*S*). In contrast, the stereoselectivity at C-4 could sometimes be altered by changing the enzyme variant used.

**Fig. 3:**
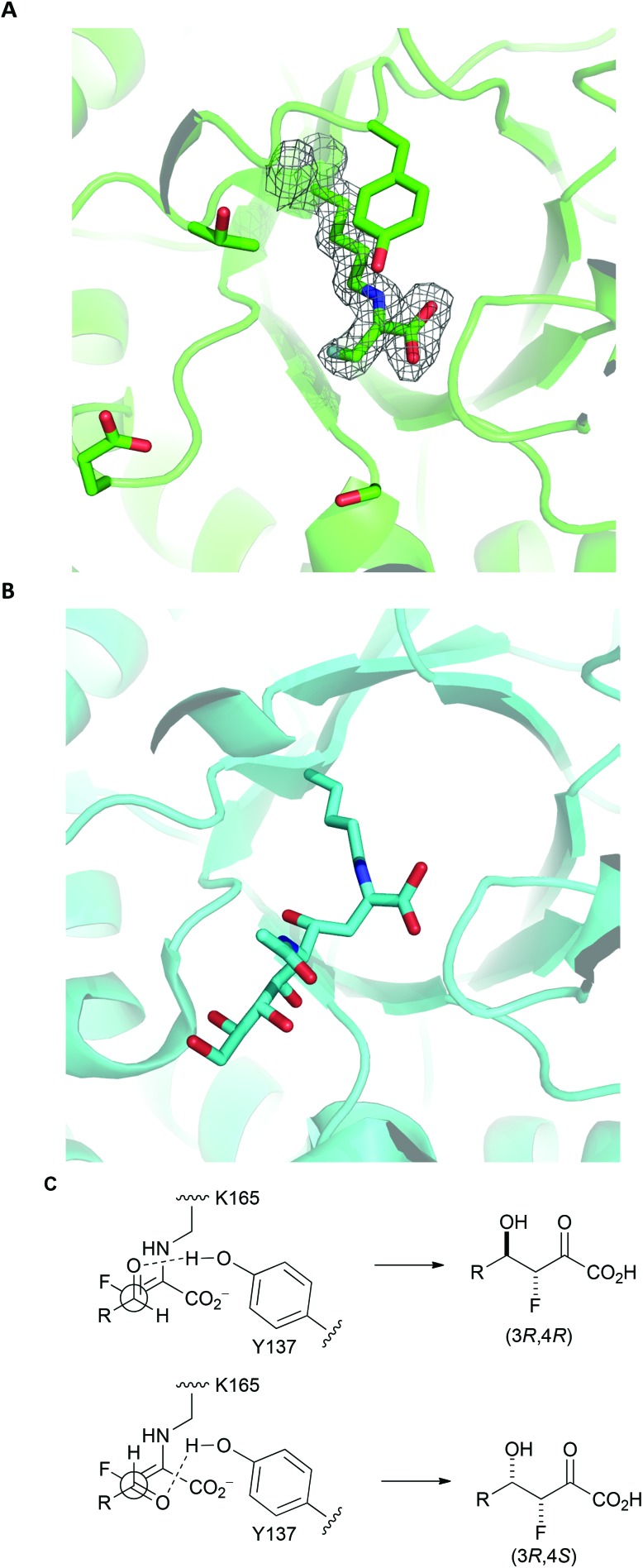
Rationale for stereoselectivity of NAL-catalysed reactions between fluoropyruvate and aldehyde substrates. Panel A: *S. aureus* NAL in complex with fluoropyruvate with general acid Y137 and residues that have key roles in recognition and stereocontrol (T167, E192 and S208) shown (PDB: ; 5A8G). The top face (as depicted) of the *Z*-configured enamine is poised to react with an aldehyde substrate. Panel B: Y137A variant of *E. coli* NAL in compex with 4-*epi*-Neu5Ac (PDB: ; 4BWL). Panel C: Possible stereochemical outcomes of the reaction of the top face of the *Z*-configured enamine with an aldehyde substrate.

To gain an insight into the structural basis of stereoselectivity, the crystal structure of *S. aureus* NAL was determined in complex with fluoropyruvate (PDB: ; 5A8G) (Panel A, Fig. 3); the structure and kinetic properties of *S. aureus* NAL have been previously shown to be extremely similar to those of *E. coli* NAL.^22^ The formation of a *Z*-configured enamine was observed, which presents only one face to aldehyde substrates. Reaction of this face of the (*Z*)-enamine intermediate would necessarily lead to the formation of 3*R*-configured products. The structure^10^ of an aldol product (4-*epi*-Neu5Ac) in complex with NAL (the Y137A variant of the *E. coli* enzyme) (PDB: ; 4BWL) is provided for comparison (Panel B, Fig. 3). Previous studies have shown that an analogue of **DHOB** – (2*R*,3*S*)-2,3-trihydroxy-4-oxo-*N*,*N*-dipropyl butanamide – can bind to the E192N variant of *E. coli* NAL in two distinct conformations (Panel B, Fig. 3).^15^ Aldehyde substrates may react *via* conformations that allow protonation by the general acid Y137.^10^ The facial selectivity of the reaction of the aldehyde determines the configuration of the product – (3*R*,4*R*) or (3*R*,4*S*) – obtained (Panel C, Fig. 3). In some cases, the ratio of C-4 epimers could be changed by altering the specific NAL variant used.

## Conclusions

NAL variants can be useful catalysts of reactions between fluoropyruvate and aldehyde substrates. Wild-type NAL catalysed the reaction between fluoropyruvate and ManNAc, albeit much less efficiently than with pyruvate as donor. It was shown that a 90 : 10 ratio of (3*R*,4*R*)- and (3*S*,4*R*)-configured products was obtained under kinetic control; whilst a 30 : 70 mixture of these products was obtained at equilibrium. The switch between kinetic and thermodynamic control may account for previous apparently conflicting reports of the outcome of this reaction.^12^

It was also shown that NAL variants are useful catalysts of reactions between fluoropyruvate and unnatural aldehyde substrates. The efficiency of catalysis varied widely, depending on the specific combination of NAL variant and aldehyde used. However, using the aldehyde **DHOB** or its enantiomer as substrate, three of the four possible diastereomeric products could be isolated.

It was noted that, under kinetic control, all productive NAL variant-catalysed reactions involving fluoropyruvate yielded (3*R*)-configured products selectively. The crystal structure of *S. aureus* NAL in complex with fluoropyruvate reveals the presence of a (*Z*)-configured enamine. The (3*R*)-selectivity of NAL catalysed reactions may be rationalised in terms of selective reaction of this (*Z*)-configured enamine *via* the face that is presented to aldehyde substrates.
