# Supplementary material for: Evaluation of fluoropyruvate as nucleophile in reactions catalysed by N-acetyl neuraminic acid lyase variants: scope, limitations and stereoselectivity
Source: Org Biomol Chem. 2015 Nov 5;14(1):105–12. doi: 10.1039/c5ob02037a (PMC4717870; doi:10.1039/c5ob02037a)
Supplement: Supplementary file 1 [file OB-014-C5OB02037A-s001.pdf]

Electronic Supplementary Material (ESI) for Organic & Biomolecular Chemistry.
This journal is © The Royal Society of Chemistry 2015

Evaluation of fluoropyruvate as nucleophile in reactions catalysed by *N*-acetyl neuraminic acid lyase variants: Scope, limitations and stereoselectivity

Jennifer Stockwell, Adam Daniels, Claire L. Windle, Thomas Harman, Thomas Woodhall, Tomas Lebl, Chi H. Trinh, Keith Mulholland, Arwen R. Pearson, Alan Berry* and Adam Nelson*

Supporting Information

Contents

S1. Biological materials and methods 2

S2. Preparation and characterisation of compounds. 9

S3. References 21

S1. Biological Materials and Methods

2×TY Bacterial growth medium

Bacterial cultures were grown in 2 TY medium. One litre of 2×TY medium contains 16 g tryptone, 10 g yeast extract and 5 g NaCl.

Enzymes

L-Lactate dehydrogenase from rabbit muscle was purchased from Roche, Mannheim, Germany.

Chromatographic media

Chelating Sepharose Fast Flow™ resin charged with Ni^{2+} was used for purification of His-tagged NAL and was purchased from Amersham Biosciences, Buckinghamshire, UK. Resins were stored in ethanol in a 50 ml Falcon tube without Ni^{2+} bound and were activated by decanting off the ethanol, washing with water then washing buffer. A 0.2M $NiCl_2$ solution is then poured onto the resin which is placed on a roller at 4 °C for 1 hour. The $NiCl_2$ solution is then decanted off and the Ni^{2+} activated is then washed twice with water then washing buffer.

Aseptic technique

Standard aseptic techniques were used throughout. Sterilisation of media and heat resistant materials was carried out using an autoclave. Heat labile solutions were sterilised by filtration through 0.22 µm MiniSart® filters (Sartourius AG, Goettingen, Germany).

Determination of pH

The pH of buffer solutions was determined using a Jenway 3020 pH meter, calibrated according to the manufacturer's instructions.

Spectrophotometry

Absorbances were measured using a Kontron Instruments UVIKON 930 spectrophotometer.

Centrifugation

Centrifugation was performed using a Beckman Coulter Avanti® J-26 XP Series high-performance centrifuge. Unless otherwise stated, centrifugation was performed at 4 °C.

Cell lyses

Cell lyses was carried out using a cell disruptor supplied by Constant Cell Disruption System, Nothants UK. The cells were lysed at 20 kpsi.

Dialysis

NAL samples were dialysed with dialysis tubing against 50-100 times the volume of the relevant buffer for two 8-16 hr periods at 4 °C. Dialysis tubing (12-14 kDa molecular weight cut-off) was purchased from Medicell International Ltd., London, UK.

Buffers

Protein purification loading/washing buffer:

50 mM Tris-HCl pH 7.4

20 mM imidazole

0.5 M NaCl

Protein purification elution buffer:

50 mM Tris-HCl pH 7.4

0.5 M imidazole

0.5 M NaCl

Dialysis buffer:

Tris: 50 mM Tris-HCl pH 7.4, 50 mM NaCl

Ammonium acetate: 20 mM ammonium acetate pH 7.4

Purification of His Tagged NAL

E. coli cells expressing NAL E192N mutant were grown in day cultures containing 5 ml 2xYT media, 5µl glycerol solution containing the *E. coli* spores and 5 µl of 100 mg/ml ampicillin solution for 6-8 hr at 37 °C. Day cultures were then transferred to night cultures which were grown at 37 °C in 2×YT media supplemented with 50 µg/mL ampicillin and 0.1 mM IPTG. The

cells were harvested by centrifugation (9 000 g, 20 mins) and the pellet was re-suspended in washing buffer using a homogeniser. The cells were lysed and the cell debris was collected by centrifugation (30 000 g, 45 mins). The supernatant was loaded onto chelating sepharose resin (pre-equilibrated with the wash buffer), in a 50 mL Falcon tube. The suspension was placed on a roller for 1 hr. The suspension was then centrifuged (4000 g, 5 mins, 4 °C) the supernatant was removed, washing buffer added (30 ml) and the suspension placed on a roller for 15 mins followed by centrifugation (4000 g, 5 mins). Contaminating proteins were removed by washing the resin a further 3 times with washing buffer (roll for 15 mins, followed by centrifugation at 4000 g for 5 mins). Elution buffer was added to the resin and placed on a roller for 1 hr. The suspension was centrifuged (4000 g, 5 mins) and the eluted His tagged NAL enzyme dissolved in the supernatant was decanted from the resin. The resin was then washed for a second time with elution buffer and rolled (15 mins) followed by centrifugation (4000 g, 5 mins) and the supernatant was collected. The eluted NAL was then dialysed (12 hr, 4 °C) Tris-HCl dialysis buffer. The following morning, the dialysis tubes containing eluted NAL were then transferred to fresh dialysis buffer (50 mM Tris/HCl, 50 mM NaCl, pH 7.5) and left to dialyse (4 hr, 4 °C). The dialysed solution was then sterile filtered into falcon tubes and stored at 4 °C. For longer-term storage, the NAL was dialysed into ammonium acetate buffer and freeze-dried. Freeze-dried protein was re-dissolved into a suitable buffer depending on the experiment required.

SDS Page

Protein purity was determined by SDS page. The composition of the running gel and stacking gel are given in Table S1. The ladder was provided by Fermentas and the gel was stained (Methanol (50% v/v), acetic acid (10% v/v), Coomassie Brilliant Blue (0.25% v/v) and Water (39.75% v/v)) and destained (Methanol (50% v/v), acetic acid (10% v/v), and Water (40% v/v)).

Table S1: Composition of gels for SDS page

Gel	Component	Quantity (µl)
Running	Acrylamide (30%)	7500
	1.5 M Tris-HCl	3750
	SDS (10%)	150
	Water	3500
	APS (Ammonium persulfate) (50 mg in 200 µl) (25%)	50
	TEMED (Tetramethylethylenediamine)	5
Stacking	Acrylamide (30%)	625
	1.5 M Tris-HCl	625
	SDS (10%)	50
	Water	3650
	APS (Ammonium persulfate) (50 mg in 200 µl) (25%)	50
	TEMED (Tetramethylethylenediamine)	5

Measurement of concentration of NAL

Concentration was determined by transferring 900 µl 100 mM Tris.HCl buffer (pH 7.4) into a 1 ml quartz cuvette (path length 1 cm) as a blank to calibrate the spectrometer. 100 µl dialysed NAL solution was added to the buffer and the absorbance measured at 280 nm. The extinction coefficient of His_6-tagged NAL is 24870 $M^{-1}cm^{-1}$, which according to the Beer-Lambert Law gives a protein concentration of 1 mg/ml when A_{280} = 0.743.[1]

Concentration of NAL

Concentration of NAL solutions were carried out using 15 ml centrifuge filters (Regenerated cellulose 10 000 NMWL) purchased from Amicon Ultra – IS. Centrifuge filters were prepared by washing with water, followed by centrifugation (2187 g, 10 min) then three times with buffer (depending on which buffer the protein to be concentrated is dissolved into) followed by centrifugation (2187 g, 10 min). The protein solution was then transferred to the filter and centrifuged (2187 g) until the desired volume/concentration was achieve.

Monitoring of reaction between fluoropyruvate and ManNAc catalysed by wild-type NAL

^{19}F NMR time course experiments were performed at 37 °C in a glass NMR tube containing 20 mM Tris-HCl pH 7.4, 100 mM aldehyde substrate (ManNAc), 20 mM sodium 3-fluoropyruvate, 10% (v/v) D_2O and a suitable aliquot of NAL. Reaction mixtures were assembled by the addition of each reaction component, with the exception of NAL. The reaction was initiated by adding NAL solution and mixing thoroughly. ^{19}F NMR spectra were recorded (Figure S1) at 15 min intervals for 1 hr; then 1 hr intervals for 23 hr; and then 12 hr intevals for 48 hr. Thereafter, individual ^{19}F NMR spectra were recorded every few days, with fresh enzyme added to restore activity.

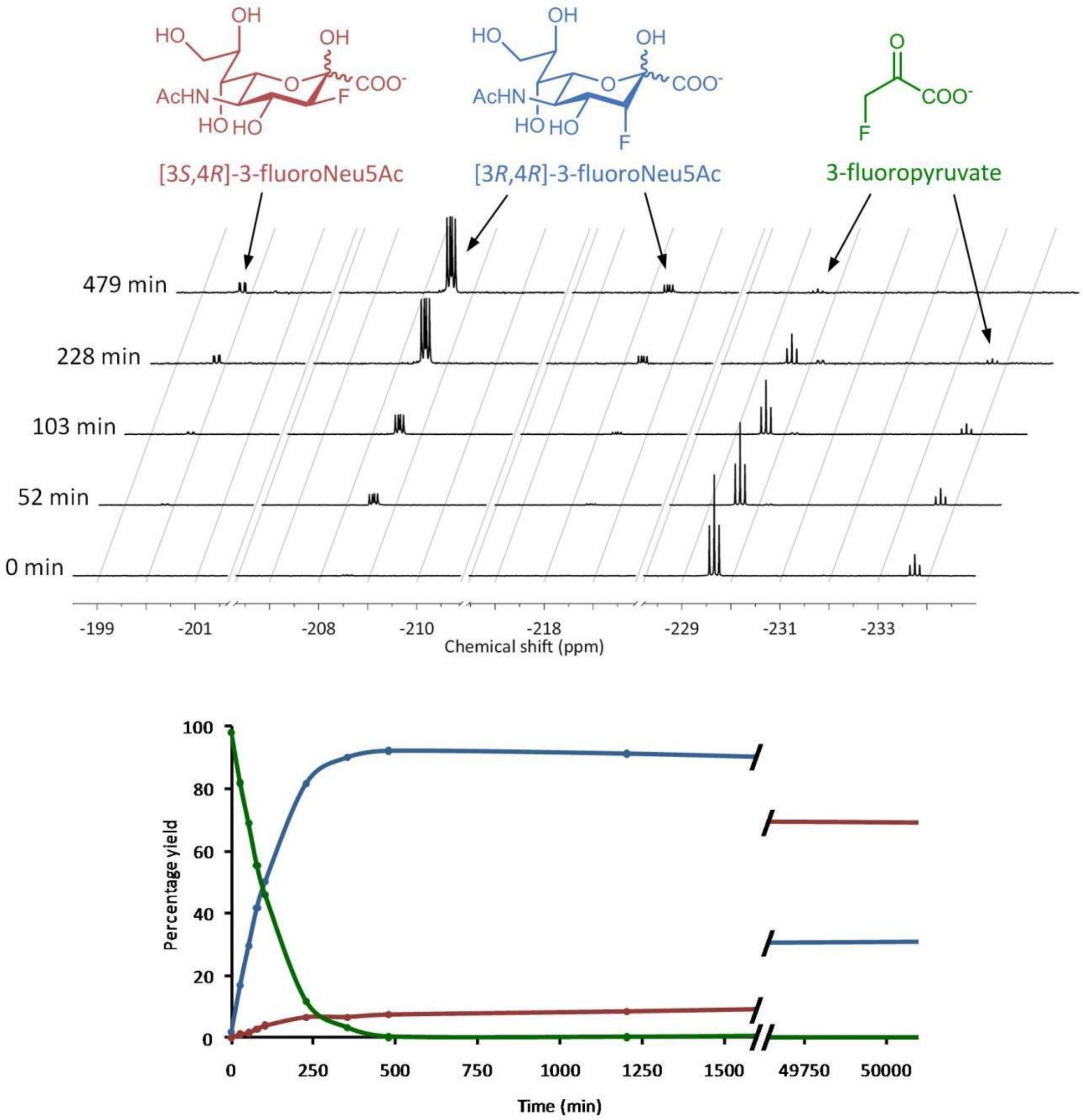

Figure S1. Time course of the wild-type ecNAL-catalysed reaction between ManNAc (100 mM) and 3-fluoropyruvate (20 mM) performed at 37 °C, pH 7.4. Top: ^{19}F NMR spectra as a function of time. Bottom: Components of the reaction in which 3-fluoropyruvate (green) depletes, and diastereomeric products are produced (blue and red). The reaction reached thermodynamic equilibrium after approximately 50,000 minutes.

Monitoring reactions catalysed by NAL variants

For each experiment 0.1 mmol of alkene was cleaved by standard ozonolysis conditions. After quenching the solution was transferred to an NMR tube and concentrated by blowing nitrogen over the solution to remove all volatiles. The NMR tubes were then placed in a drying tube on the high-vacuum line over-night to remove all trace of volatile contaminants. A 1 M solution of sodium fluoropyruvate in 50 mM Tris-HCl buffer pH 7.4 (20 µl, 0.02 mmol) was added followed by NAL in 50 mM Tris-HCl buffer pH 7.4 (980 µl) (NAL concentrations: E192N, 1.00 mg/ml; E192N/T167V/S208V, 0.90 mg/ml; E192N/T167G, 1.82 mg/ml). A sealed capillary tube containing deuterium oxide was placed inside the NMR tube. The experiment was kept at room temperature and re-submitted for 296 MHz ^{19}F NMR initially at 1 hr intervals for the first 7 hr, then at 3-6 hr intervals thereafter up to 36 hr. Conversions were calculated by comparing the relative integrals of the product peaks to the fluoropyruvate peaks. When the fluoropyruvate peaks were no longer visible in the NMR spectrum, the reaction was judged to have gone to completion.

Protein crystallisation and complex production

Wild-type *sa*NAL crystals were produced using the following conditions: 100mM Tris/HCl (pH 7.0-8.5), 200 mM NaCl, polyethylene glycol (PEG) 3350 (16-28% wt/vol). Crystals were grown by hanging drop vapour diffusion and yielded crystals in 7-10 days. The enzyme-fluoropyruvate complex was produced by soaking the wild-type *sa*NAL crystals in mother liquor supplemented with fluoropyruvate (100 mM) and 15% (v/v) PEG 400 for 1 min before being sequentially transferred to mother liquor with 5% increments in PEG 400 concentration. The final soak contained the mother liquor supplemented with fluoropyruvate (100 mM) and 25%(v/v) PEG 400. Crystals were then flash-cooled in liquid nitrogen prior to data collection.

Data collection and refinement

Data collection was carried out on beamline I04 at Diamond Light Source. The data set was collected from a single crystal at 100 K. Integration and scaling of data was carried out by XDS[2] and SCALA.[3,4] The structure of the wild-type saNAL enzyme in complex with fluoropyruvate was solved by molecular replacement in Phaser using the structure of wild-type saNAL (PDB ID:4AHP) as the search model. REFMAC5[5] was used for refinement of

the data and after each refinement cycle model building was performed in COOT.[6] Coordinates and restraint library files for the lysine residue covalently bound to fluoropyruvate (HETcode: KPF) were generated using the PRODRG server and were manually edited.[7] The model was validated using the Molprobity server.[8]

Table S2: Structural data statistics for the X-ray crystallographic structure of wild-type *sa*NAL in complex with fluoropyruvate

	Diamond beamline	I04
	PBD accession code	5a8g
	Space group	$C222_1$
	a (Å)	62.59
	b (Å)	150.40
	c (Å)	140.28
	R_{merge}[a,b]	0.086 (0.516)
	R_{pim}[b,c]	0.031 (0.189)
	Observed reflections	594439
	Unique reflections	70045
	Completeness (%)[b]	98.7 (91.4)
	Multiplicityb	8.5 (8.0)
	<I/σ> I/σ [b]	14.1 (3.4)
Refinement	R_{factor} (%)	0.1811
	R_{free} (%)[d]	0.2114
	No. of protein atoms	4643
	No. of solvent molecules	346
	No of ligand atoms	12
	Average overall B-factor (Å^2)	24.1
	Average ligand B-factor (Å^2)	29.1
	RMS bond lengths (Å)[e]	0.013
	RMS bond angles (Å) ξ	1.64
Ramachandran analysis[f]	Most favoured	98.78
	Outliers	1

[a]$R_{merge} = \Sigma_{hkl}\Sigma_i|I_i(hkl) - \langle I(hkl)\rangle| / \Sigma_{hkl}\Sigma_i|I_i(hkl)$. [b]Values given in parentheses correspond to those in the outermost shell of the resolution range. [c]Rpim –precision indicating (multiplicity-weighted) Rmerge, relative to all I+ or I-. [d]R_{free} was calculated with 5% of the reflections set aside randomly. [e]Based on the ideal geometry values of Engh & Huber (ref. 9). [f]Ramachandran analysis using the program MolProbity (ref. 10). The percentage of residues in the regions of the plot is indicated. The side-chain of Tyr111 is in close proximity to Leu142, Thr143 and Phe110 from an adjacent chain causing the phi and psi angles of Tyr111 to lie in an unfavoured region of the Ramachandran plot.

S2. Preparation and characterisation of compounds

General Experimental

All non-aqueous reactions were performed under an atmosphere of nitrogen unless otherwise stated. Water-sensitive reactions were performed in oven-dried glassware, cooled under nitrogen before use. Solvents were removed *in vacuo* using a Büchi rotary evaporator and a Vacuubrand PC2001 Vario diaphragm pump. Tetrahydrofuran (THF), CH_2Cl_2, toluene and CH_3CN were dried and purified by means of a Pure Solv MD solvent purification system (Innovative Technology Inc.). Anhydrous *N,N*-dimethylformamide (DMF) was obtained in SureSeal bottles from Sigma-Aldrich. All other solvents used were of chromatography or analytical grade. Petrol refers to petroleum spirit (b.p. 40-60 °C). Commercially available starting materials were obtained from Sigma-Aldrich, Fluka, Acros or Alfa-Aesar and were used without purification unless stated. Ozone was generated using a Welsbach generator at 0.4-0.6 psi.

Thin layer chromatography (TLC) was carried out on aluminium backed silica (Merck silica gel 60 F_{254}) plates supplied by Merck. Visualisation of the plates was achieved using an ultraviolet lamp (λ_{max} = 254 nm), $KMnO_4$, anisaldehyde or ninhydrin. LCMS analysis was generally carried out on an Agilent 1200 series LC system comprising a Bruker HCT Ultra ion trap mass spectrometer; he solvent system used was CH_3CN/H_2O + 0.1% formic acid with a Phenomenex Luna C18 50 × 2 mm 5 micron column.

Flash chromatography was carried out using silica gel (35-70 µm particles) supplied by Merck. Ion exchange chromatography was carried out using Dowex® 1×8 200-400 resin or Discovery SAX pre-packed cartridges. Mass-directed HPLC purification was carried out using an Agilent 1260 Infinity HPLC system comprising an Agilent 6120 Quadrupole LC/MS and Agilent G1968D active splitter. Semi preparative HPLC was carried out on an Algilent Technologies 1200 series instrument using a reverse-phase C18 Hyperclone column with a gradient elution 0:100 → 20:80 acetonitrile–TFA (0.1% v/v):water–TFA (0.1% v/v) over 30 min, a flow rate of 3.0 ml/min.

Optical rotation measurements were carried out at the sodium D-line (589 nm) on a Schmidt and Haensch H532 or an Optical Activity AA-1000 polarimeter instrument; concentrations are g/100 mL, temperatures given in °C, optical rotations are given in 10^{-}

^{1}degcm^{2}g^{-1} (units are omitted). Melting points were recorded on a Reichert hot stage microscope. Infrared spectra were recorded on a Perkin-Elmer One FT-IR spectrometer with absorption reported in wavenumbers (cm^{-1}). High resolution mass spectra (HRMS) were recorded on a Bruker Daltonics micrOTOF or Bruker MaXis Impact spectrometer with electrospray ionisation (ESI) source; where EI ionisation was required, a Waters/Micromass GCT Premier spectrometer was used.

Proton (^{1}H), fluorine (^{19}F) and carbon (^{13}C) NMR spectral data were collected on Bruker Avance DPX 300, Avance 500 or DRX 500 spectrophotometers and the spectra were processed using MestReNova NMR processing software. Chemical shifts (δ) are quoted in parts per million (ppm) downfield of tetramethylsilane and referenced to the residual solvent peak. Coupling constants (*J*) are quoted in Hertz (Hz) and splitting patterns reported in an abbreviated manner: app. (apparent), s (singlet), d (doublet), t (triplet), q (quartet), m (multiplet). Assignments were made with the aid of COSY, DEPT-135, HMQC, HMBC and NOESY experiments. The ^{1}H/19F-HSQC-TOCSY experiment was acquired using *hsqcdietgpsi* pulse sequence from the Bruker pulse sequence library.

General method for ozonolysis

Unless otherwise stated, ozonolysis was carried out under the following conditions. The alkene was dissolved in methanol (0.1 mmol of alkene per 0.5 ml of MeOH), cooled to – 78°C, purged with O_2 for at least 10 min and then exposed to ozone. Once a blue colour was observed, excess ozone was purged from the reaction with O_2. Me_2S (0.15 ml per 0.1 mmol of alkene) was added and the reaction was allowed to stir under nitrogen until all peroxides were quenched (starch-iodide paper).

Preparation of Specific Compounds

General procedure for enzymatic syntheses with wild-type NAL

N-Acetyl-D-mannosamine (1.11 g, 5 mmol) and sodium 3-fluoro-pyruvate (128 mg, 1 mmol) were dissolved in 100 mM Tris-HCl buffer pH 7.4 (46.05 mL). Wild-type ecNAL solution (3.95 mL, 17 mg) was added and the reaction incubated at 37 °C for 24 h (for the isolation of **4a**) or >1 week (with regular addition of fresh enzyme, for the isolation of **4d**). The reaction mixture was concentrated under reduced pressure to give a crude product which was

purified by flash chromatography, eluting with 4:2 EtOAc–MeOH then 4:2:0.2 EtOAc–MeOH–H_2O.

(3*S*,4*R*)-5-Acetamido-3,5-dideoxy-3-fluoro-D-*erythro*-L-*gluco*-2-nonulopyranosonic acid 4d

The general procedure gave after >1 week the fluorinated sialic acid mimetic[11] **4d** (13 mg, 43%; 96:4 mixture of anomers) as a colourless film, R_f 0.3 (4:2:1.5:0.1 EtOAc–MeOH–H_2O–AcOH); [*α*]D –56.7 (*c*. 1.00 in H2O); Major anomer ^{1}H NMR (500 MHz, D_2O) δ 4.47 (1H, dd, $^2J_{HF}$ 49.7 and $^3J_{HH}$ 8.8 Hz, H-3), 3.97-3.84 (3H, m, H-4, 5 and 6), 3.65 (1H, dd, *J* 11.8 and 2.2 Hz, H-9_A), 3.57-3.52 (1H, m, H-8), 3.43 (1H, dd, *J* 11.8 and 6.4 Hz, H-9_B), 3.32 (1H, d, *J* 9.2 Hz, H-7), 1.88 (3H, s, acetyl Me); Major anomer ^{19}F-NMR (470 MHz, 90% H_2O, 10% D_2O) δ –199.3 (dd, *J* 49.7 and 12.0 Hz); *m/z* (ES–) 326.

(3*R*,4*R*)-Acetamido-3,5-dideoxy-3-fluoro-D-*erythro*-L-*manno*-2-nonulopyranosonic acid 4a

The general procedure gave after 24 hr the fluorinated sialic acid mimetic[11] **4a** (103 mg, 34%, 98:2 mixture of anomers) as amorphous crystals, R_f 0.2 (4:2:1.5:0.1 EtOAc–MeOH–H_2O–AcOH); [*α*]D –16.0 (*c*. 1.00 in H_2O); Major anomer ^{1}H NMR (500 MHz, D_2O) δ 4.65 (1H, d, $^2J_{HF}$ 49.3 Hz, H-3), 4.07 (1H, app. t, *J* 10.6 Hz, H-5), 3.93 (1H, ddd, $^3J_{HF}$ 30.0 and *J* 10.6 and 2.1 Hz, H-4), 3.87 (1H, d, *J* 10.6 Hz, H-6), 3.71-3.66 (2H, m, H-8 and 9_A), 3.46 (1H, dd, *J* 12.5 and 7.1 Hz, H-9_B), 3.34 (1H, d, *J* 8.9 Hz, H-7), 1.87 (3H, s, acetyl Me); ^{19}F NMR (470 MHz, 90% H_2O, 10% D_2O) δ –208.1 (dd, *J* 49.3 and 30.0 Hz, major anomer), –217.9 (dd, *J* 51.3 and 29.9 Hz, minor anomer); *m/z* (ES–) 326.

2,3-*O*-Isopropylidene-D-lyxono-1,4-lactone 6

Concentrated sulfuric acid (3.75 mL), was added to a solution of lactone[12] **5** (4.6 g, 31 mmol) in acetone (200 mL) and stirred under nitrogen for 8 hr. The mixture was neutralised with solid sodium carbonate, filtered through celite and the filtrate dried ($MgSO_4$), filtered and concentred under reduced pressure. The residue was purified by flash chromatography, eluting with 50 : 50 ethyl acetate–petrol to give the lactone **6** as a colourless micro needles (2.6 g, 44%), m.p. 96.3-99.7 °C (from EtOAc–Petrol); R_f: 0.3 (70:30, EtOAc–Petrol); $[\alpha]_D^{22}$: 63.8 (c. 1.3, Acetone) [lit. 90.3 (c. 1.0, Acetone)]; υ_{max} /cm^{-1} (film) 3210, 2998, 1772; δH

(500 MHz; $CDCl_3$) 4.92-4.85 (2H, m, 2- and 3-H), 4.63 (1H, ddd, *J* 6.8, 5.2 and 3.4, 4-H), 4.00 (2H, ddd, *J* 16.4, 11.7 and 6.4, 5-H), 2.36 (1H, br s, 5-OH), 1.49 (3H, s, Me), 1.40 (3H, s, Me); δC (75 MHz; $CDCl_3$) 173.0, 114.0, 78.5, 75.6, 75.5, 60.4, 25.6, 23.6; *m/z* (LCMS ES+) [$M+Na^+$] 211 (45%, $M+Na^+$).

5-Deoxy-5-iodo-2,3-O-isopropylidene-D-lyxono-1,4-lactone 7

Triphenylphosphine (11 g, 42 mmol) and imidazole (2.6 g, 38 mmol) were added to a solution of the lactone **6** (2.6 g, 14 mmol) in toluene (30 mL). The mixture was heated to 70 °C, then iodine (8 g, 63 mmol) was added in portions. After 30 min the reaction was allowed to cool to room temperature, was quenched with sodium thoisulfate solution (1 M, 500 mL) and extracted with ethyl acetate (4 × 500 mL). The combined organic fractions were washed with brine (250 mL), dried ($MgSO_4$), filtered and concentrated under reduced pressure. The residue was purified by flash chromatography eluting with 5 :95 ethyl acetate�petrol, then 20:80 ethyl acetate–petrol to give the iodide **7** as a yellow oil (3.98 g, 96%) . m.p. 95.9-99.2 °C (EtOAc–Petrol); R_f: 0.35 (2:8, EtOAc–Petrol); $[\alpha]_D^{21}$: −22.9 (c. 1.2, CH_2Cl_2); υ_{max} /cm^{-1} (solid) 2990, 1782, 1190; δ_H (500 MHz; $CDCl_3$) 5.00 (1H, d, *J* 6.4, 3-H), 4.65-4.60 (2H, m, 2- and 4-H), 3.46 (1H, dd, *J* 9.8 and 4.6, 5-H_A), 3.43 (1H, dd, *J* 9.7 and 5.3, 5-H_A), 1.47 (3H, s, Me), 1.40 (3H, s, Me); δC (75 MHz; $CDCl_3$) 165.1, 134.2, 130.6, 60.5, 48.7, 22.1, 20.0, 13.4; *m/z* (ES^+) [$M+H^+$] 299 (100%).

(2*S*, 3*S*)-2,3-*O*-Isopropylidene-pent-4-eonic acid *ent*-8

Zinc/copper couple (7.1 g) was added to a solution of iodide **7** (3.98 g, 13 mmol) in acetone–water (4 : 1, 33 mL) and the mixture was refluxed for 2 h, then allowed to cool to room temperature. The mixture was filtered through Celite, concentrated under reduced pressure, and then redissolved in chloroform (40 mL) containing formic acid (1 mL). The mixture was washed with brine (25 mL), the aqueous phase was extracted with chloroform (4 × 25 mL), the combined organics were dried ($MgSO_4$), filtered and concentrated under reduced pressure to give the acid *ent*-**8** (2.05 g, 89%) as a colourless oil, R_f: 0.8 (1:99, AcOH:EtOAc); $[\alpha]_D^{24}$: 32.3 (c. 1.3, $CHCl_3$), spectroscopically identical to the enantiomeric compound.[13]

(2*S*, 3*S*)-2,3-Isopropylidene-pent-4-enoic acid dipropylamide *ent*-9

Dipropylamine (2.6 mL, 19 mmol), 1-hydroxybenzotriazole (2.35 g, 17 mmol) and 1-(3-dimethylaminopropyl)-3-ethylcarbodiimide hydrocholride (3.6 g, 23 mmol) were added to a solution of the acid *ent*-**8** (2 g, 11.6 mmol) in ethyl acetate (200 mL) and stirred under nitrogen at room temperature for 18 hr. Water (250 mL) and ethyl acetate (200 mL) were added, the aqueous phase was extracted with ethyl acetate (3 × 250 mL), dried ($MgSO_4$), filtered and evaporated under reduced pressure. The residue was purified by flash chromatography, eluting with 80:20 petrol–ethyl acetate to give the dipropylamide *ent*-**9** as (2 g, 59%) as a colourless oil. R_f: 0.2 (80:20, Petrol–EtOAc); $[\alpha]_D^{25}$: 37.8 (c. 0.9, $CHCl_3$) [lit.[13] −28.6 (c. 0.91, $CHCl_3$) for the enantiomeric compound], spectroscopically identical to the enantiomeric compound.[3]

(2*S*, 3*S*)-2,3-Dihydroxy-pent-4enoic acid dipropylamide *ent*-10

Trifluoroacetic acid–water (9:1, 155 mL) was added to the amide *ent*-**9** (2 g, 8 mmol), after 5 min the mixture was evaporated under reduced pressure and purified by flash chromatography, eluting with 60:40 petrol–ethyl acetate to give a colourless solid, recystallation with hexane gave the dipropylamide *ent*-**10** (560 mg, 33%) as colourless needles. m.p. 76.7-77.8 °C (Petrol–EtOAc); R_f: 0.2 (60:40, Petrol–EtOAc); $[\alpha]_D^{22}$: −26.7 (c. 1.2, $CHCl_3$), spectroscopically identical to the enantiomeric compound.[3]

4-Nitro-*N*-[(2*E*)-3-phenylprop-2-en-1-ylidene]benzene-1-sulfonamide 12

Boron trifluoro diethyletherate (194 µl, 1.58 mmol) was added to a refluxing solution of 4-nitrobenzenesulfonamide (4.00 g, 19.8 mmol) and cinnamaldehyde (2.49 ml, 19.8 mmol) in dry toluene (113 ml). **Caution: Effervescence occurs on addition of boron trifluoro diethyletherate.** The solution was heated under reflux for 3 days, cooled to room temperature and concentrated *in vacuo*. The residue was triturated from EtOAc to give the *imine* **12** (5.50 g, 88%) as a dark brown solid; R_F: 0.3 (20:80, EtOAc–petrol); δ_H (500 MHz; $CDCl_3$) 8.86 (1H, d, *J* 9.5, 1-H), 8.38 (2H, d, *J* 8.9, 2 × nosyl 3-H), 8.18 (2H, d, *J* 8.9, 2 × nosyl 3-H), 7.62-7.57 (3H, m, 2 × phenyl 3-H and 3-H), 7.51-7.42 (3H, m, 2 × phenyl 2-H and phenyl 4-H), 7.01 (1H, dd, J 15.8, 9.5, 2-H); δ_C (75 MHz; $CDCl_3$) 173.1 (1-C), 156.0 (3-C), 150.5 (nosyl-1-C or nosyl 4-C), 144.4 (nosyl-1-C or nosyl 4-C), 133.9 (phenyl 1-C), 132.3 (phenyl 4-C),

129.3 (nosyl 2-C or nosyl 3-C), 129.2 (2 × phenyl 2-C), 129.0 (2 × phenyl 3-C), 124.34 (nosyl 2-C or nosyl 3-C), 124.31 (2-C); HRMS Found: 317.0592, $[MH]^+$ requires 317.0591.

***N*-[(1'*R*,2'*E*)-1'-[(2*R*,5*S*,6*S*)-5,6-Dimethoxy-5,6-dimethyl-3-oxo-1,4-dioxan-2-yl]-3'-phenylprop-2'-en-1'-yl]-4-nitrobenzene1'-sulfonamide 13**

Lithium hexamethyldisilazide (1.0 M in THF, 2.4 ml, 2.4 mmol) was added to a solution of (5*S*,6*S*)-5,6-dimethoxy-5,6-dimethyl-1,4-dioxan-2-one[14] (300 mg, 1.6 mmol) in THF (5 ml) at –78 °C and stirred for 15 min. A solution of the imine **12** (500 mg, 1.6 mmol) in THF (10 ml) was added and the reaction stirred for 1 hr at –78 °C before being quenched with acetic acid (0.4 ml) and filtered through a short plug of silica, eluting with ether. The solvents were removed *in vacuo* and the resulting crude material was purified by flash column chromatography (20:80 EtOAc–petrol). The excess *p*-nosyl sulfonamide was removed by dissolving the residue in chloroform, filtering under vacuum and concentrating the filtrate *in vacuo* to give the *lactone* **13** (643 mg, 79%, d.r. 94:6) as a sticky yellow-orange solid; R_F: 0.43 (1:1, petrol–EtOAc); $[\alpha]_D^{22}$: 92.9 (c. 0.50 in chloroform); υ_{max}/cm^{-1} (film) 3271, 3106, 2951, 1748, 1530, 1450; δ_H (500 MHz; $CDCl_3$) 8.15 (2H, d, *J* 8.8, 2 × nosyl 2-H), 7.98 (2H, d, *J* 8.7, 2 × nosyl 3-H), 7.23-7.20 (3H, m, 2 × phenyl 3-H and phenyl 4-H), 7.09-7.05 (2H, m, 2 × phenyl 2-H), 6.32 (1H, d, *J* 16.0, 3-H'), 5.82 (1H, d, *J* 7.2, N*H*), 5.80 (1H, dd, *J* 16.0 and 9.2, 2-H'), 4.55-4.49 (1H, m, 1-H'), 4.39 (1H, d, *J* 3.1, 2-H), 3.32 (3H, s, OMe_A), 3.20 (3H, s, OMe_B), 1.45 (3H, s, Me_A), 1.43 (3H, s, Me_B); δ_C (75 MHz; $CDCl_3$) 165.9 (3-C'), 149.7 (nosyl 4-C), 146.4 (nosyl 1-C), 135.2 (phenyl 1-C), 135.1 (3-C'), 128.8 (2 × nosyl 3-C), 128.6 (2 × phenyl 3-C and phenyl 4-C), 126.3 (2 × phenyl 2-C), 124.1 (2 × nosyl 2-C), 122.3 (2-C'), 105.4 (6-C), 98.6 (5-C), 73.6 (2-C), 58.6 (1-C'), 50.2 (OMe_A), 49.5 (OMe_B), 17.7 (Me_A), 16.8 (Me_B); HRMS Found: 529.1260, $[MNa]^+$ requires 529.1251. The diastereomeric ratio was determined by the relative integration of the peaks at 4.55-4.49 (1H, m, 1-H'$_{maj}$) and 4.68-4.60 (1H, m, 1-H'$_{min}$) in the 500 MHz ^{1}H NMR spectrum.

Ent-**13** was prepared on a 6 g scale in 91% yield from (5*R*,6*R*)-5,6-dimethoxy-5,6-dimethyl-1,4-dioxan-2-one,[4] $[\alpha]_D^{22}$: −39.8 (c. 1.30 in chloroform).

(2*R*,3*R*,4*E*)-2-Hydroxyl-3-[(4-nitrobenzene)sulfonamide]-5-phenyl-*N*,*N*-dipropylpent-4-enamide 14

Trimethylaluminium (2.00 M in hexane, 1.0 ml, 2.00 mmol) was added to a solution of dipropylamine (273 µl, 2.00 mmol) in dry CH_2Cl_2 (5 ml) and stirred at room temperature for 15 min. The lactone **13** (0.50 g, 1.00 mmol) was dissolved in the minimum amount of CH_2Cl_2 and added to the stirred solution of Me_2AlNPr_2 and the mixture was warmed to 40 °C, stirred for 2 hr, cooled to room temperature and quench by addition of 1 M $HCl_{(aq)}$ (1 ml). **Caution: addition of HCl results in a vigorous effervescent reaction.** The reaction mixture was extracted with CH_2Cl_2 (3 × 30 ml), dried (Na_2SO_4) and concentrated *in vacuo*. The residue was dissolved in 1:1 MeCN–water (50 ml) to which TFA (1.2 ml) was added and the mixture stirred at room temperature for 24 hr, poured into EtOAc, separated and the aqueous layer extracted with EtOAc (2 × 30 ml). Combined organic layers were dried ($MgSO_4$) and concentrated *in vacuo*. Purification by flash column chromatography (30:70 EtOAc–petrol) to give *dipropylamide* **14** (324 mg, 88%) as a dark orange/yellow solid; R_F: 0.37 (1:1, petrol-EtOAc); m.p. 121-123 °C (petrol–EtOAc); $[\alpha]_D^{22}$: 45 (c. 0.80 in chloroform); υ_{max}/cm^{-1} (film) 3203, 2969, 1645, 1532; δ_H (500 MHz; $CDCl_3$) 8.13 (2H, d, *J* 8.5, 2 × nosyl 2-H), 7.99 (2H, d, *J* 8.5, 2 × nosyl 3-H), 7.20 (3H, s, 2 × phenyl 2-H and phenyl 4-H), 7.05-6.96 (2H, m, 2 × phenyl 3-H), 6.12 (1H, d, *J* 15.9, 5-H), 5.64 (1H, dd, *J* 15.8 and 8.1, 4-H), 4.16 (1H, d, *J* 2.1, 2-H), 4.29 (1H, d, *J* 8.1, 3-H), 3.61-3.51 (1H, m, propyl 1-H_A), 3.37-3.27 (1H, m, propyl 1-H_B), 3.25-3.14 (1H, m, propyl-1-H_C), 3.03-2.90 (1H, m, propyl 1-H_D), 1.71-1.55 (2H, m, propyl 2-$H_{2,A}$), 1.51-1.40 (2H, m, propyl 2-$H_{2,B}$), 0.94 (3H, t, *J* 7.3, propyl 3-$H_{3,A}$), 0.82 (3H, t, *J* 7.3, propyl 3-$H_{3,B}$); δ_C (75 MHz; MeOD) 172.4 (1-C), 151.0 (nosyl 4-C), 148.8 (nosyl 1-C), 137.2 (phenyl 1-C), 135.2 (phenyl 5-C), 129.7 (2 × phenyl 2-C), 129.5 (2 × phenyl 3-C), 129.1 (phenyl 4-C), 127.3 (2 × nosyl 3-C), 125.2 (2 × nosyl 2-C), 124.2 (4-C), 72.3 (2-C), 60.7 (3-C), 50.3 (propyl 1-C_A), 49.1 (propyl 1-C_B), 23.4 (propyl 2-C_A), 21.8 (propyl 2-C_B), 11.7 (propyl 3-C_A), 11.4 (propyl 3-C_B); HRMS Found: 476.1862, $[MH]^+$ requires 476.185.

Ent-**14** was also prepared on a 2 g scale from *ent*-**13**, $[\alpha]_D^{22}$: −48.7 (c. 1.7 in chloroform).

(2*R*,3*R*,4*E*)-3-Amino-2-hydroxy-5-phenyl-*N*,*N*-dipropylpent-4-enamide S1

Thiophenol (300 µl, 2.97 mmol) and 1,8-diazabicycloundec-7-ene (600 µl, 3.96 mmol) were added to a solution of the lactone **14** (470 mg, 0.99 mmol) in MeCN (4 ml) and stirred at room temperature for 16 hr. The reaction mixture was concentrated *in vacuo* and purified by flash column chromatography (gradient elution 5:95 EtOH–CH_2Cl_2 → 5:5:90 sat. NH_3 in MeOH–EtOH–CH_2Cl_2) to give the *amino alcohol* **S1** (255 mg, 89%) as a pale yellow viscous

oil; R_F: 0.18 (5:95, EtOH–CH_2Cl_2); $[\alpha]_D^{22}$: 55.1 (c. 0.90 in chloroform); υ_{max}/cm^{-1} (film) 3277, 2965, 1638; δ_H (500 MHz; $CDCl_3$) 7.34-7.20 (5H, m, 2 × phenyl 2-H, 2 × phenyl 3-H and phenyl 4-H), 6.52 (1H, d, *J* 15.9, 5-H), 6.12 (1H, dd, *J* 15.9 and 7.9, 4-H), 4.60 (1H, d, *J* 3.2, 2-H), 3.88 (2H, s, NH_2), 3.82 (1H, dd, *J* 7.7 and 2.8, 3-H), 3.60-3.51 (1H, m, propyl 1-H_A), 3.39-3.31 (1H, m, propyl 1-H_B), 3.20-3.12 (1H, m, propyl 1-H_C), 3.01-2.93 (1H, m, propyl-1-H_D), 1.63-1.54 (2H, m, propyl 2-H_{2A}), 1.52-1.45 (2H, m, propyl 2-H_{2B}), 0.90 (3H, t, *J* 7.4, propyl-3-H_{3A}), 0.93 (3H, t, *J* 7.4, propyl 3-H_{3B}); δ_C (75 MHz; $CDCl_3$) 171.0 (1-C), 133.2 (5-C), 128.9 (phenyl 1-C), 128.5 (2 × phenyl 2-C), 127.9 (phenyl 4-C), 126.6 (2 × phenyl 3-C), 126.0 (4-C), 70.2 (2-C), 56.8 (3-C), 48.8 (propyl 1-C_A), 47.6 (propyl 1-C_B), 22.1 (propyl 2-C_A), 20.7 (propyl 2-C_B), 11.4 (propyl 3-C_A), 11.1 (propyl 3-C_B); HRMS Found: 291.2067, $[MH]^+$ requires 291.2067.

Ent-**S1** was also prepared on a 1 g scale from *ent*-**14**, $[\alpha]_D^{22}$: −30.5 (c. 1.2 in chloroform).

(2*R*,3*R*,4*E*)-3-Acetamido-2-hydroxy-5-phenyl-*N*,*N*-dipropylpent-4-enamide 15

A solution of the amino alcohol **S1** (225 mg, 0.77 mmol), $NaHCO_3$ (520 mg, 6.16 mmol) and acetic anhydride (0.11 ml, 1.16 mmol) in methanol (5 ml) was heated under reflux for 1 hr before being cooled to room temperature, filtered and concentrated *in vacuo*. Purification by flash column chromatography (80:20 EtOAc–petrol) to give the *dipropylamide* **15** (228 mg, 89%) as a pale yellow/colourless amorphous solid; R_F: 0.16 (80:20, EtOAc–petrol); $[\alpha]_D^{22}$: 29.1 (c. 1.30 in chloroform); υ_{max}/cm^{-1} (film) 3234, 1628; δ_H (500 MHz; $CDCl_3$) 7.32-7.27 (4H, m, 2 × phenyl 2-H and 2 × phenyl 3-H), 7.24-7.20 (1H, m, phenyl 4-H), 6.53 (1H, d, *J* 15.9, 5-H), 6.36 (1H, d, *J* 8.4, N*H*), 6.05 (1H, dd, *J* 15.9 and 7.2, 4-H), 4.85-4.81 (1H, m, 3-H), 4.59 (1H, d, *J* 2.2, 2-H), 3.68-3.56 (2H, m, propyl 1-$H_{2,A}$), 3.30-3.23 (1H, m, propyl 1-H_B), 3.01-2.94 (1H, m, propyl 1-H_C), 2.06 (3H, s, acetyl-CH_3), 1.70-1.60 (2H, m, propyl 2-$H_{2,A}$), 1.57-1.47 (2H, m, propyl 2-$H_{2,B}$), 0.97 (3H, t, *J* 7.4, propyl 3-$H_{3,A}$), 0.85 (3H, t, *J* 7.4, propyl 3-$H_{3,B}$); δ_C (75 MHz; $CDCl_3$) 170.4 (amide-C=O), 169.8 (acetyl-C=O), 136.2 (phenyl 1-C), 134.0 (5-C), 128.6 (2 × phenyl 2-C), 128.0 (phenyl 4-C), 126.6 (2 × phenyl 3-C), 122.5 (4-C), 70.3 (2-C), 53.7 (3-C), 48.4 (propyl 1-C_A), 47.5 (propyl 1-C_B), 23.4 (acetyl-CH_3), 22.1 (propyl 2-C_A), 20.7 (propyl 2-C_B), 11.4 (propyl 3-C_A), 11.0 (propyl 3-C_B); HRMS Found: 333.2165, $[MH]^+$ requires 333.2173.

Ent-**15** was also prepared on a 1 g scale from *ent*-**S1**, $[\alpha]_D^{22}$: −34.4 (c. 0.9 in chloroform).

General method for enzymatic synthesis of fluorinated dipropylamides

The alkene was cleaved under standard ozonolysis conditions (see **General method**). Methanol was removed *in vacuo* and the crude mixture was re-dissolved in 50 mM tris buffer (1.2 ml per 0.1 mmol aldehyde) to which sodium fluoropyruvate (0.5-1 eq.) was added. The pH was adjusted to 7.4 by addition of NaOH (2 M) followed by addition of NAL variant (in 50 mM tris buffer, 2-4 mg per 0.1 mmol aldehyde). The reaction was allowed to stir under nitrogen for 24 hr. The mixture was frozen, thawed and filtered through Celite® and the product was isolated by ion exchange chromatrography using Dowex® resin (gradient elution 0 → 2 M MeO_2H in water) or SAX resin cartridges (gradient elution 0 → 2 M MeO_2H in MeOH).

(3*R*,4*S*,5*S*,6*R*)-7-(Dipropylamino)-3-fluoro-4,5,6-trihydroxy-2,7-dioxoheptanoate 16c

By the general method, the alkene **10** (222 mg, 1.03 mmol), sodium fluoropyruvate (88 mg, 0.69 mmol) and the E192N/T167V/S208V NAL variant gave a crude product was purified by ion exchange chromatography and reverse-phase HPLC (retention time: 31.0 min) to give the *fluorinated sialic acid analogue* **16c** (90 mg, 41%) as a yellow glass, δ_H (500 MHz, D_2O) 5.03 (1H, dd, $^2J_{HF}$ 52.0, $^3J_{HH}$ 5.5, 3-H^{min,pyran}), 4.86 (1H, dd, $^2J_{HF}$ 48.1, $^3J_{HH}$ 7.3, H-3^{min,furan}), 4.85 (1H, dd, $^2J_{HF}$ 49.7, $^3J_{HH}$ 4.8, 3-H^{maj,pyran}), 4.72 (1H, d, $^2J_{HF}$ 44.2, H-3^{maj,furan}), 4.65 (1H, d, $^3J_{HH}$ 6.1, 6-H^{maj,pyran}), 4.57 (1H, d, $^3J_{HH}$ 7.2, 6-H^{min,pyran}), 4.46 (1H, dd, $^3J_{HF}$ 18.7, $^3J_{HH}$ 5.6, 4-H^{min,pyran}), 4.39 (1H, dd, $^3J_{HF}$ 22.4, $^3J_{HH}$ 3.7, 4-H^{maj,pyran}), 4.30 (1H, td, $^3J_{HH}$ 7.7 and 3.4, H-4^{min,furan}), 4.18 (1H, dd, $^3J_{HH}$ 8.6 and 5.1, H-5^{min,furan}), 4.12 (1H, t, $^3J_{HH}$ 5.0, 5-H^{maj,pyran}), 4.02 (1H, dd, $^3J_{HH}$ 10.4 and 7.4, H-4^{maj,furan}), 3.96 (1H, dd, $^3J_{HH}$ 10.0 and 4.5, H-5^{maj,furan}), 3.95 (1H, dd, $^3J_{HH}$ 6.4, 5-H^{min,pyran}); 3.43-2.99 (4H, m, 2 × propyl 1-H_2), 1.58-1.37 (4H, m, 2 × propyl 2-H_2), 0.99-0.66 (6H, m, 2 × propyl 3-H_3); δ_F (395 MHz, D_2O) –190.5 (d, $^2J_{HF}$ 50.5, $^3J_{HF}$ 24.0, 3-F^{maj,pyran}), –194.5 (d, $^2J_{HF}$ 43.7, $^3J_{HF}$ 4.7, 3-F^{maj,furan}), –201.9 (d, $^2J_{HF}$ 53.1, $^3J_{HF}$ 18.7, 3-F^{min,pyran}), –207.4 (d, $^2J_{HF}$ 48.5, $^3J_{HF}$ 10.1, 3-F^{min,furan}); *m/z* (ES) $[MH]^+$ 324.1 (100 % $[MH]^+$). The fluorinated sialic acid analogue **16c** was observed as a 35:25:30:10 mixture of major pyranose, minor pyranose, major furanose and minor furanose forms. The ratio of species was determined analysis of the 395 MHz ^{19}F NMR spectrum. The ^1H NMR spectra of the species were indirectly extracted using $^{19}F/^1H$ HSQC-TOCSY (Figure S2).

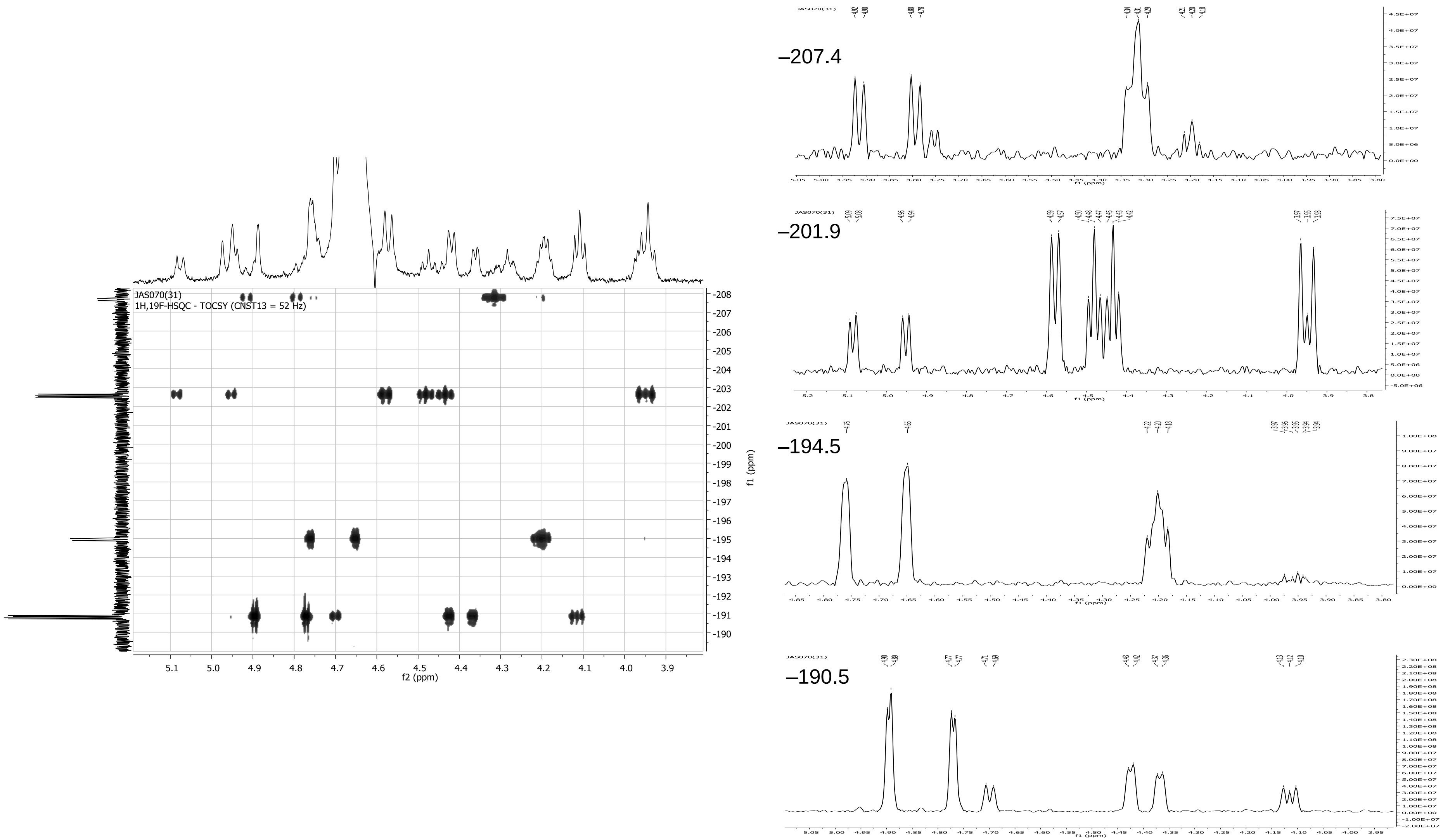

Figure S2: Spectra of the fluorinated sialic acid analogue **16c**. Left: $^{1}H/^{19}F$-HSQC-TOCSY spectrum. Right: Extracted ^{1}H NMR spectra for each of the anomeric pyranose/furanose forms.

(3*R*,4*S*,5*S*,6*R*) and (3*R*,4*R*,5*S*,6*R*)-7-(Dipropylamino)-3-fluoro-4,5,6-trihydroxy-2,7-dioxoheptanoate 16c and 16a

By the general method, the alkene **10** (314 mg, 1.58 mmol), sodium fluoropyruuvate (202 mg, 1.58 mmol) and the E192N NAL variant gave a crude product which was purified by ion exchange chromatography the *fluorinated sialic acid analogues* **16c** and **16a** as a yellow glass (171 mg, 33%, **16c**:**16a** 60:40). Purification by reverse-phase HPLC (**16a**: retention time, 28.7 min; **16c**: retention time, 31.0 min) gave small samples of the individual diastereoisomers. The spectroscopic data for major diastereomer **16c** was identical to that previously obtained. The minor diastereomer **16a** existed as a 92:8 mixture of pyranose forms, δ_H (500 MHz, D_2O) 4.78 (1H, d, $^2J_{HF}$ 49.9, 3-H), 4.57 (1H, d, $^3J_{HH}$ 9.2, 6-H), 3.97-3.90 (1H, m, 4-H), 3.88 (1H, t, $^2J_{HH}$ 9.7, 5-H), 3.42-3.08 (4H, m, 2 × propyl 1-H_2), 1.58-1.38 (4H, m, 2 × propyl 2-H_2), 0.78-0.68 (6H, m, 2 × propyl 3-H_3); δ_F (395 MHz, D_2O) –206 (1F, dd, $^2J_{HF}$ 49.9, $^3J_{HF}$ 32.5, 3-F); *m/z* (ES) $[MH]^+$ 324.1 (100 % $[MH]^+$).

(3R,4S,5R,6S)-7-(Dipropylamino)-3-fluoro-4,5,6-trihydroxy-2,7-dioxoheptanoate *ent*-16d

By the general method, the alkene *ent*-**10** (202 mg, 0.93 mmol), sodium fluoropyruvate (119 mg, 0.93 mmol) and the E192N/T167V/S208V NAL variant to give a crude product was purified by ion exchange chromatography to give the *fluorinated sialic acid analogue ent*-**16d** (155 mg, 52%, d.r. >98:<2) as a colourless glass which existed as a single pyranose form, δ_H (500 MHz, D_2O) 4.62 (1H, d, $^3J_{HH}$ 9.7, 6-H), 4.60 (1H, dd, $^2J_{HF}$ 49.3 $^3J_{HH}$ 9.3, 3-H), 3.95 (1H, dt, $^3J_{HF}$ 13.3 $^3J_{HH}$ 9.4, 4-H), 3.78 (1H, t, $^2J_{HH}$ 9.5, 5-H), 3.42-3.12 (4H, m, 2 × propyl 1-H_2), 1.60-1.39 (4H, m, 2 × propyl 2-H_2), 0.83-0.70 (6H, m, 2 × propyl 3-H_3); δ_F (395 MHz, D_2O) –199.8 (1F, dd, $^2J_{HF}$ 49.3, $^3J_{HF}$ 13.3, 3-F); *m/z* (ES) $[MH]^+$ 324.1 (100 % $[MH]^+$).

(3*R*,4*R*,5*S*,6*R*)-7-(dipropylamino)-5-acetamido-3-fluoro-4,6-dihydroxy-2,7-dioxoheptanoate 17a

By the general method, the alkene **15** (100 mg, 0.3 mmol), sodium fluoropyruuvate (38 mg, 0.3 mmol) and the E192N NAL variant gave a crude cruduct which was purified by ion exchange chromatography and mass-directed HPLC to give the *fluorinated N-acetyl dipropylamide sialic acid derivative* **17a** (5.3 mg, 7%, 98:2 mixture of diastereomers). The product **17a** existed as a 98:2 mixture of pyranose anomers, δ_H (500 MHz; D_2O) 4.95 (1H, dd, $^2J_{HF}$ 49.0, $^3J_{HH}$ 2.1, 3-H^{min}), 4.90 (1H, dd, $^2J_{HF}$ 49.2, $^3J_{HH}$ 2.2, 3-H^{maj}), 4.84 (1H, d, $^3J_{HH}$ 10, 6-H^{min}), 4.83 (1H, d, $^3J_{HH}$ 10, 6-H^{maj}), 4.23 (1H, t, $^3J_{HH}$ 9.9, 5-H^{maj}), 4.22 (1H, t, $^3J_{HH}$ 1.9, 5-H^{min}), 4.17 (1H, ddd, $^3J_{HF}$ 29.1, $^3J_{HH}$ 10.9 and 2.2, 4-H^{min}), 4.16 (1H, ddd, $^3J_{HF}$ 29.1, $^3J_{HH}$ 10.9 and 2.2, 4-H^{maj}), 3.55-3.43 (2H, m, propyl 1-$H_{2,A}$), 3.25-3.18 (1H, m, propyl 1-H_C), 3.00-2.93 (1H, m, propyl 1-H_D), 1.91 (3H, s, acetyl-CH_3), 1.62-1.51 (2H, m, propyl 2-H_A), 1.48-1.39 (2H, m, propyl 2-H_A), 0.81 (3H, t, $^3J_{HH}$ 7.7, propyl

3-H_A), 0.78 (3H, t, $^3J_{HH}$ 7.5, propyl 3-H_A); δ_C (75 MHz; D_2O) 174.1 (acetyl-C=O), 170.8 (1-C), 168.7 (7-C), 95.4 (2-C), 89.7 (d, $^1J_{CF}$ 178.0, 3-C), 67.6 (6-C), 66.9 (5-C), 66.8 (4-C), 49.8 (propyl 1-C_A), 48.7 (propyl 1-C_B), 22.1 (propyl 2-C_A), 21.9 (acetyl-CH_3), 20.1 (propyl 2-C_A), 10.5 (propyl 3-C_B), 10.3 (propyl 3-C_A); δ_F (296 MHz, D_2O) –199.2 (1F, dd, $^2J_{HF}$ 49.3, $^3J_{HF}$ 10.4, 3-F^{3S4R}), –207.8 (1F, dd, $^2J_{HF}$ 49.0, $^3J_{HF}$ 29.1, 3-$F^{3R4R,min}$), –218.5 (1F, dd, $^2J_{HF}$ 50.2, $^3J_{HF}$ 28.8, 3-$F^{3R4R,maj}$). HRMS Found: 363.1565, [M]-H^+ requires 363.1573.

S3. References

1. M. R. Wilkins, E. Gasteiger, A. Bairoch, J.-C. Sanchez, K. L. Williams, R. D. Appel and D. F. Hochstrasser, in *Methods in Molecular Biology; 2-D proteome analysis protocols,* Humana Press Inc, 1999, vol. 112, p. 531.
2. W. Kabsch, *Acta Crystallogr. D Biol. Crystallogr.* 2010, **66,** 125.
3. A. G. W. Leslie, *Acta Crystallogr. D Biol. Crystallogr.* 2006, **62,** 48.
4. P. Evans, *Acta Crystallogr. D Biol. Crystallogr.* 2006, **62,** 72.
5. G. N. Murshudov, P. Skubak, A. A. Lebedev, N. S. Pannu, R. A. Steiner, R. A. Nicholls, M. D. Winn, F. Long and A. A. Vagin, *Acta Crystallogr. D Biol. Crystallogr.* 2011, **67,** 355.
6. P. Emsley, B. Lohkamp, W. G. Scott and K. Cowtan, *Acta Crystallogr. D Biol. Crystallogr.* 2010, **66,** 486.
7. A. W. Schuttelkopf and D. M. F. Van Aalten, *Acta Crystallogr. D Biol. Crystallogr.* 2004, **60,** 1355.
8. V. B. Chen, W. B. Arendall, J. J. Headd, D. A. Keedy, R. M. Immormino, G. J. Kapral, L. W. Murray, J. S. Richardson and D. C. Richardson, *Acta Crystallogr. D Biol. Crystallogr.* 2010, **66,** 12.
9. R. A. Engh and R. Huber, *Acta Crystallogr. A* 1991, **47,** 392.
10. S. C. Lovell, I. W. Davis, W. B. Adrendall, P. I. W. De Bakker, J. M. Word, M. G. Prisant, J. S. Richardson and D. C. Richardson *Proteins-Structure Function and Genetics* 2003, **50,** 437.
11. H. A. Chokhawala, H. Cao, H. Yu and X. Chen, *J. Am. Chem. Soc.* 2007, **129**, 10630.
12. S.-Y. Han, M. M. Joullie, V. V. Fokin and N. A. Petasis, *Tetrahedron Asymm.* 1994, **5**, 2535.
13. T. Woodhall, G. J. Williams, A. Berry and A. Nelson, *Org. Biomol. Chem.* 2005, **3**, 1795.
14. S. V. Ley, E. Diez, D. Dixon, R. T. Guy, P. Michel, G. L. Nattrass and T. D. Sheppard, *Org. Biomol. Chem.* 2004, **2**, 3608.
